# Characterising the spatial distribution of opportunities and constraints for land sparing in Brazil

**DOI:** 10.1038/s41598-020-58770-5

**Published:** 2020-02-06

**Authors:** Juliana Silveira dos Santos, Rafael Feltran-Barbieri, Ellen S. Fonte, Andrew Balmford, Veronica Maioli, Agnieszka Latawiec, Bernardo B. N. Strassburg, Benjamin T. Phalan

**Affiliations:** 1 International Institute of Sustainability, Rio de Janeiro, 22460-320 Brazil; 2 Ecology Department, Spatial Ecology and Conservation Lab (LEEC), São Paulo State University, UNESP, Avenida 24 A, 1515 Bela Vista, Rio Claro, São Paulo Brazil; 3 Laboratório de Genética & Biodiversidade, Instituto de Ciências Biológicas, Universidade Federal de Goiás, 74690-900 Goiânia, Goiás Brazil; 4 World Resources Institute, Washington, DC 20002 USA; 5 Department of Zoology, University of Cambridge, Cambridge, CB2 3EJ UK; 6 Rio Conservation and Sustainability Science Centre, Department of Geography and the Environment, Pontifícia Universidade Católica, 22453900 Rio de Janeiro, Brazil; 7 Institute of Agricultural Engineering and Informatics, Faculty of Production and Power Engineering, University of Agriculture in Kraków, Balicka 116B, 30-149 Kraków, Poland; 8 School of Environmental Sciences, University of East Anglia, Norwich, NR4 7TJ UK; 9 Programa de Pós Graduação em Ecologia, Universidade Federal do Rio de Janeiro, 68020 Rio de Janeiro, Brazil; 10 Botanical Garden Research Institute of Rio de Janeiro, 22460-030 Rio de Janeiro, Brazil; 11 Instituto de Biologia, Universidade Federal da Bahia, Salvador, Bahia 40170-115 Brazil; 12 Parque das Aves, Foz do Iguaçu, Paraná 85855-750 Brazil

**Keywords:** Biodiversity, Environmental impact

## Abstract

Brazil is a megadiversity country with more tropical forest than any other, and is a leading agricultural producer. The technical potential to reconcile these roles by concentrating agriculture on existing farmland and sparing land for nature is well-established, but the spatial overlap of this potential with conservation priorities and institutional constraints remains poorly understood. We mapped conservation priorities, food production potential and socio-economic variables likely to influence the success of land sparing. Pasture occupies 70% of agricultural land but contributes ≤11% of the domestic food supply. Increasing yields on pasture would add little to Brazil’s food supply but – if combined with concerted conservation and restoration policies – provides the greatest opportunities for reducing land demand. Our study illustrates a method for identifying municipalities where land-sparing policies are most likely to succeed, and those where further effort is needed to overcome constraints such as land tenure insecurity, lack of access to technical advice, labour constraints, and non-compliance with environmental law.

## Introduction

Continuing to produce food while averting the mass extinction of biodiversity are two of the greatest challenges facing humanity this century. One promising approach to address both is to protect and restore native vegetation, while increasing yields to minimize the area required for croplands and pasture^1^. In broad terms, the biophysical and technical potential to spare land for nature in this way is well-established^2^. This is the case in Brazil, a megadiversity country which supports more tropical forest than any other, and which is among the world’s leading producers of beef, soybeans, sugarcane and other commodities. Increasing yields of pasturelands in Brazil to half of their potential would release enough cultivated land to meet even the highest demand scenarios for meat, crops, wood products and biofuels until at least 2040, without further loss of native vegetation^3^. Less is known, however, about how the potential for both production and conservation are distributed in relation to each other across the country, and in relation to social and economic conditions conducive to land sparing^4^.

Land-sparing policies must deliver two concurrent outcomes – conserving native vegetation and increasing yields – and to do so will typically need to create linkages between these outcomes. Such policies are more likely to succeed when certain enabling conditions are in place (Table 1)^5^. Adoption of more sustainable, high-yielding methods will be facilitated by access to extension services, availability of sufficient skilled labour and land tenure security. However, attempts to implement land sparing are vulnerable to rebound effects, where some of the land savings of yield increases are cancelled out by greater incentives for agricultural expansion^6^. Backfire, when yield increases accelerate agricultural expansion, is also a possibility. Rebounds and backfire can occur if high-yielding farming is more profitable, where market demand is relatively elastic, and where there is inadequate protection of conservation land. A related risk is that of leakage, where increased protection of native vegetation fails to reduce deforestation overall, but merely displaces it^6^. This can occur if conservation measures are not linked to measures to increase yields and concentrate agricultural production on a smaller area. Coordinated conservation and agricultural policies are essential to avert the risks of rebounds, backfire and leakage^1^.

**Table 1:** Selected institutional variables, and how they are expected to influence the success of land-sparing policies.

Variable	Expected influence on success of land sparing	Mapped constraint
Technical advice	Where available: greater potential for adoption and dissemination of technologies and practices that can increase yields, avoid land degradation and improve compliance with environmental legislation. Where unavailable: less potential for dissemination of new practices and technologies; such areas could be targeted to improve knowledge exchange through farmer networks or civil society support.	Percentage of those receiving technical advice is below median (25.3%)
Labour availability	Where high: potential to increase yields using labour-intensive methods, but also greater risk of leakage if practices used to increase yields reduce the need for labour. Where low: less risk of leakage, but perhaps more challenging and costly to close yield gaps; targeted technical advice could help.	Very high or very low labour availability (within upper or lower quartiles)
Land tenure security	Where secure (with legal title or lease): farmers can have confidence they will not lose investments in soils, irrigation equipment and other investments in productivity; also likely to have greater access to credit and more interest in improving yields. Where insecure (without legal title): farmers have little incentive to focus on sustaining yields in long term, more incentive to maximize current-year profits even if it results in land degradation; such areas could be targeted for efforts to strengthen and formalize land tenure.	Percentage of those without secure tenure is above median (2.1%)
Size of rural properties	Where high: farmers have greater access to credit and capital to invest in yield increases, and must comply with Forest Code; fewer farmers may be eligible for agricultural support from government. Where low: smallholders have access to agricultural support, but may have less access to credit or private capital and may struggle to close yield gaps (which may not be a priority for them); fewer opportunities for large-scale conservation/restoration within single landholdings; opportunities to improve access to technical support, credit and to develop landowner networks for conservation.	*Not mapped as constraint as all property size classes present both opportunities and constraints*
Educational attainment	Where high: local actors may have greater agency, such as ability to access and adopt new practices and to participate in planning and policy processes related to land use and conservation. Where low: poor educational outcomes may be an impediment to local agency, and could impede inclusion of farmers in planning and policy processes related to land use and conservation, in the absence of concerted efforts to enable and improve communication and participation.	Percentage of adults who completed at least middle school is below median (20.15%)
Type of product and market	Where staple food crops, destined to local markets: price elasticity of demand relatively low, so rebound effects likely to be less pronounced. Where non-food crops and luxury crops destined to export markets: price elasticity of demand higher, and thus greater potential for rebound effects that undermine land sparing.	*Incorporated into alternative estimate of production potential*
Forest Code deficit	Where low (high compliance with Forest Code): more likely to retain native vegetation on private lands. Where high (low compliance with Forest Code): are less likely to retain native vegetation on private lands, but could provide funds for compensatory conservation or restoration (through tradeable forest certificates) if non-compliant landowners are obliged to meet legal requirements.	Forest Code deficit in upper quartile and comprises ≥10% of native vegetation
Forest Code surplus	Where no surplus exists: remaining native vegetation on private land (with some exceptions) is not legally available for clearance, but may still be in need of improved protection and restoration. Where surplus exists: native vegetation is vulnerable to legal clearance, but could be protected in new protected areas, with new incentives, or through tradeable forest certificates.	Forest Code surplus comprises ≥10% of native vegetation in municipality

A definition of how these variables were mapped as constraints for the illustrative analysis in Fig. 5 is provided. Our expectations are based on our reading of the literature, but can be considered as a set of preliminary conclusions or hypotheses amenable to further testing. We define success here as protection of a larger area of native vegetation, and concurrent concentration of food production on less land, than would be the case without land-sparing policies.

In theory, rebound effects will be less pronounced in landscapes where most production is of locally-consumed staple crops, and more pronounced with non-food commodities, export crops, luxury products, or where markets are distorted by subsidies^5^. For this reason, in our study, we estimated the production of crops and livestock products that contribute to domestic food supply in Brazil, subtracting from total production the net exports, waste, and the energy losses incurred for the portion of crops used to feed livestock. We anticipate that municipalities with high additional production potential of crops which are mostly consumed domestically are also those where rebounds from yield increases are less likely.

Technologies which are labour-intensive, capital-intensive and targeted to established farmlands are less likely to result in rebounds than those which displace labour, free up capital and reduce the cost of opening the frontier^7^. Where labour markets are local and segmented, with limited mobility, labour-demanding yield increases are likely to reduce habitat clearance^5^. Challenges differ in landscapes dominated by large properties, compared to those where most producers are smallholders. Wealthy landowners with political power may have greater ability to evade environmental laws, but may also be more vulnerable to reputational pressure. Policies to take some land out of production may be more ethically acceptable in large landholdings, while it is often smallholders who are in most need of technical and financial support. Good governance is critical, both to ensure land tenure security so farmers can invest in their land, and to make it more likely that environmental protections, such as the Brazilian Forest Code, are respected^8,9^.

Brazil’s recent history illustrates both the potential to make land sparing happen, and some of the challenges involved. The most prominent drivers of deforestation are beef, soybeans, sugarcane and tree plantations. In the decade to 2005, Brazil cleared almost 2 million ha of forest in the Amazon each year for pasture and cropland^10^. Through a mix of protected area expansion, land registration, satellite monitoring, improved enforcement of a reformed Forest Code, credit restrictions and incentives, and voluntary agreements by large companies, Amazon deforestation was reduced by >70% in the subsequent decade^11–14^. At the same time, production of soybeans and beef continued to increase, showing that with political will, backed up by effective environmental governance, deforestation can be reduced dramatically without preventing agricultural development.

This success is neither complete nor secure. Amazon deforestation has started to creep upwards again, and small-scale clearing in particular is on the rise^15,16^. The Brazilian government has proposed weakening environmental regulations to maintain the support of the bancada ruralista – senate and congressional representatives aligned with agribusiness^17^. The newly-inaugurated president (as of January 2019) has announced policies likely to undermine environmental protection further, including the transfer of authority over indigenous reserves to the Ministry of Agriculture^18^. Outside the Amazon, other biogeographic domains, most notably the Cerrado, are losing even greater areas of native vegetation^19^. Even if perfect compliance with the Forest Code could be achieved, it would leave more than 80 million ha of native vegetation on private land vulnerable to legal clearance^8,20^. Crop production is already relatively high-yielding, and so the potential to increase yields further may be limited^21^, although there is much progress to be made on promoting high-yielding practices that address social and environmental issues such as income inequality and excessive use of pesticides^22,23^. Substantial scope for improving pasture yields has been identified^3^, beyond the gains already made^24^, with the caveat that great care is needed to minimize social and environmental risks through practices such as rotational grazing and integration of livestock systems with crops and forestry^25,26^. While closing yield gaps is needed to minimize further expansion, it is unlikely to serve on its own to improve food security^27^, nor act as an effective lever for land sparing^1,28^. This is especially true given the prevalence of export crops in Brazil, for which yield increases have less effect on local prices; ~70% of both sugarcane and soybeans are exported (FAOSTAT). A range of initiatives are striving to address these complex challenges at different scales^29^.

Understanding patterns of production potential, conservation priority, and enabling conditions for land sparing could help to identify local opportunities and constraints, and assist with targeting such initiatives to where they have most chance of success. Here, we map parts of Brazil with most potential for increasing agricultural production on existing pastures and croplands, and identify areas where habitat protection and restoration are most needed. Those municipalities with both high production potential and high priority for biodiversity are likely to need municipality-level land-sparing policies. We use additional layers of information on institutional constraints to identify where such policies might be most likely to succeed. The resulting maps can be used as a first step to locate landscapes where NGO engagement with specific agricultural sectors might be most beneficial; to pinpoint where companies working towards more sustainable supply chains might focus specific efforts; to identify where government agencies could invest additional resources to enforce the provisions of the Forest Code and protect habitats; and to highlight the enabling conditions to be reinforced and the constraints to be addressed for specific programs to be implemented and scaled up.

## Results

**Figure 1:**
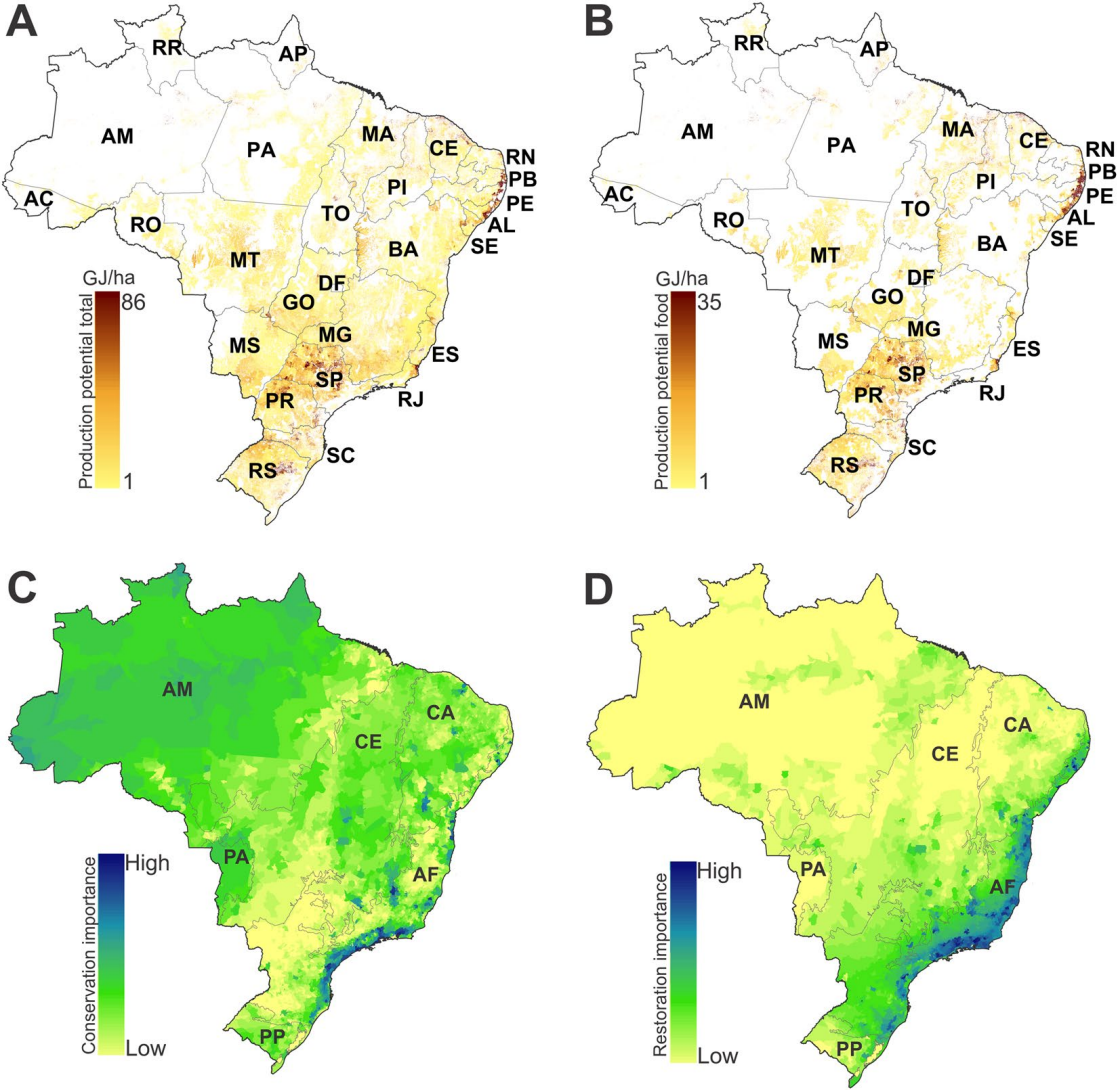
Additional production potential of seven major crops (rice, sugarcane, cassava, maize, soybean, sorghum and wheat), beef and milk on currently cultivated lands and importance of each municipality for conservation and restoration of natural vegetation. (**A**) Additional production potential in food energy (gigajoules per hectare) of all cultivated land (croplands and pasture) in each municipality. (**B**) Additional production potential adjusted to reflect contributions to domestic food production (excluding non-food uses, net exports and waste), as described in Methods. Protected areas, indigenous land and municipalities with zero additional potential are masked in white. (**C**) Importance for conservation, and (**D**) importance for restoration for terrestrial vertebrates (amphibians, birds and mammals). Conservation importance is calculated as the proportion of the remaining natural vegetation in each municipality, multiplied by the biodiversity importance. Biodiversity importance is calculated as the summed proportion of species’ ranges occurring at a location, calculated on a 1 km resolution grid, and averaged across grid cells in each municipality. Restoration importance is the proportion of cleared natural vegetation (excluding urban areas) multiplied by the biodiversity importance. Abbreviations refer to domains (AM: Amazon, PA: Pantanal, CE: Cerrado, CA: Caatinga, AF: Atlantic Forest and PP: Pampa).

The seven leading crops in Brazil contribute seven times more food energy to the domestic food supply than pastures, from one-third as much land area. These crops account for 85% of cropland in Brazil (65 out of 77 Mha). In terms of total production, including that which is exported, wasted or used for non-food purposes, the difference between cropland and pastures is even more stark, with croplands producing approximately 30 times more total food energy than Brazil’s cattle pastures. Of the cropland area, almost one-quarter (24%) is devoted to livestock feed crops. The distribution of additional production potential, combining crops and pastures, is shown in Figure 1a (total) and Figure 1b (contribution to the domestic food supply). For further details see Supplementary Figs. 1 and 2 and Supplementary Table 1.

**Figure 2:**
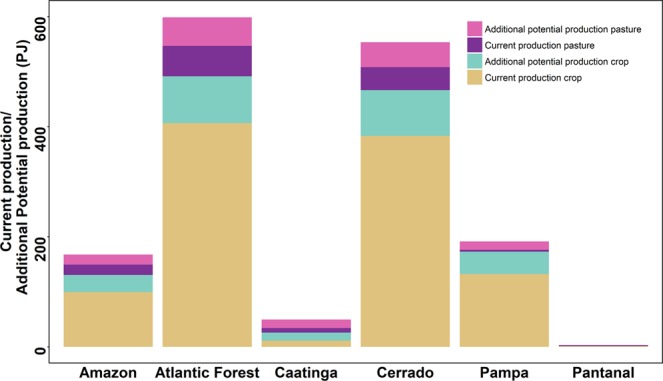
Current production and additional potential production, in petajoules of food energy, for beef and milk on pasture, and seven major crops (cassava, maize, rice, sorghum, soybean, sugarcane and wheat) on existing cropland. The greatest potential to increase food supply is on cropland, while the greatest potential to spare land for nature is on pasture. Each bar represents one biogeographic domain. For municipality-level data, see Supplementary Figs. 1–12.

While increasing crop yields could make a greater contribution to the food supply, improving yields on pastures offers greater potential to spare land for nature. In percentage terms the widest yield gaps (i.e., the difference between current yields and yields attained in other areas with similar climate^30^) are on pasture, and almost half (47%) of municipalities could double (or more) their livestock production on existing pasturelands. Overall, pasture production could be increased by 113% if current yield gaps were closed. Yield gaps in cropland were much smaller, and only 12% of municipalities could double crop production on existing croplands. Overall, crop production could be increased by 25%. However, because livestock farming is so much less productive per hectare than crop cultivation, despite the smaller area and narrower yield gaps of cropland, its additional production potential overall exceeds that of pasture (Fig. 2). For Brazil as a whole, closing yield gaps for beef and milk would increase domestic food energy supply by 13%, while closing yield gaps for the seven crops would increase it by 22%.

We also made a preliminary estimate of the additional food production possible if pastures were converted to croplands, using soybeans and sugarcane as example crops. According to Soares-Filho *et al*.^8^, ~60% of cultivated pastures in Brazil could be utilized for crop production. If the mean attainable yields for soybeans or sugarcane (whichever is greater in food energy terms) could be achieved on half this area – on 30% of cultivated pastures in each municipality – then cultivation of these crops on current pasturelands could increase domestic food supply in Brazil by 130%. Such an approach could also enable a higher economic return for farmers from a smaller area of agricultural land.

Turning to biodiversity, we mapped the importance for conservation and restoration of each municipality, using global range maps of the 901 species of amphibians, 1,899 birds and 631 mammals mapped in Brazil, and estimates of the remaining area of native vegetation. Biodiversity importance is a weighted endemism metric, calculated as the summed proportion of species’ ranges occurring at a location, calculated on a 1 km resolution grid, and averaged across grid cells in each municipality^31,32^. Important areas for conservation (defined by multiplying biodiversity importance by the proportion of remaining native vegetation) were found in all domains, with the highest values reflecting concentrations of endemic species in the Atlantic Forest, and also in the Amazon, Cerrado and Caatinga (Fig. 1C). Areas of greatest importance for restoration (high importance, but with little native vegetation) were concentrated in the Atlantic Forest, where there have been extensive losses of habitat formerly occupied by restricted-range species (Fig. 1D). Taxon-specific patterns were relatively consistent with these broad patterns, but differed in detail (see Supplementary Data).

**Figure 3:**
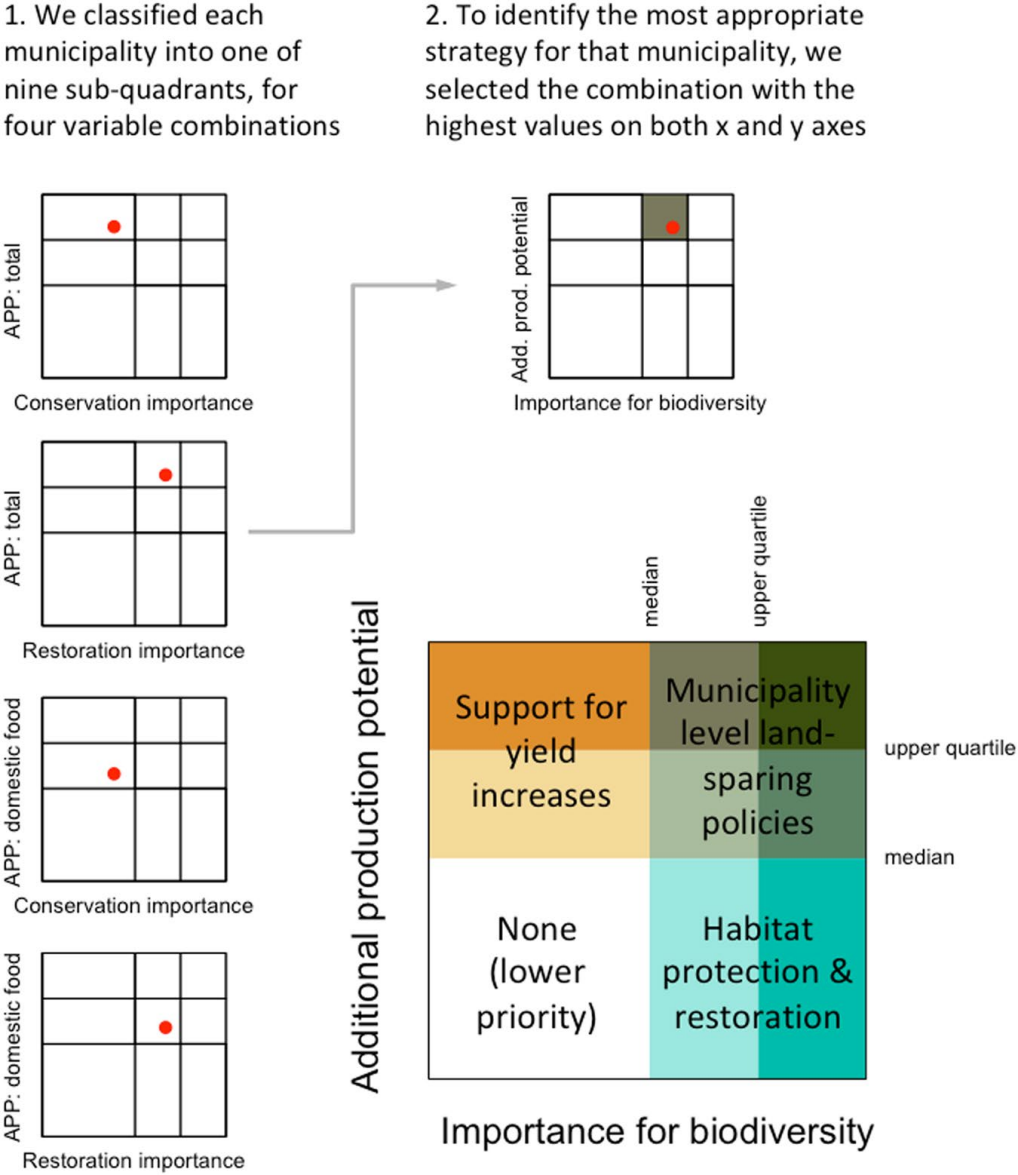
Outline of methodology for classifying municipalities into different categories according to their production potential and importance for biodiversity. In step 1, we classified each municipality (represented by the red dot) into one of nine sub-quadrants. This classification was repeated four times, for all pairwise combinations of the variables describing importance for biodiversity (restoration importance and conservation importance) and additional production potential (total and contribution to domestic food supply, expressed per unit of agricultural area). In step 2, we selected the combination with the highest values on both x and y axes. This combination was used to define the category for each municipality, as shown at bottom right. There were four main categories, defined by quadrants (support for yield increases, municipality-level land-sparing policies, habitat protection and restoration, none) and nine sub-categories (illustrated by the colors).

**Figure 4:**
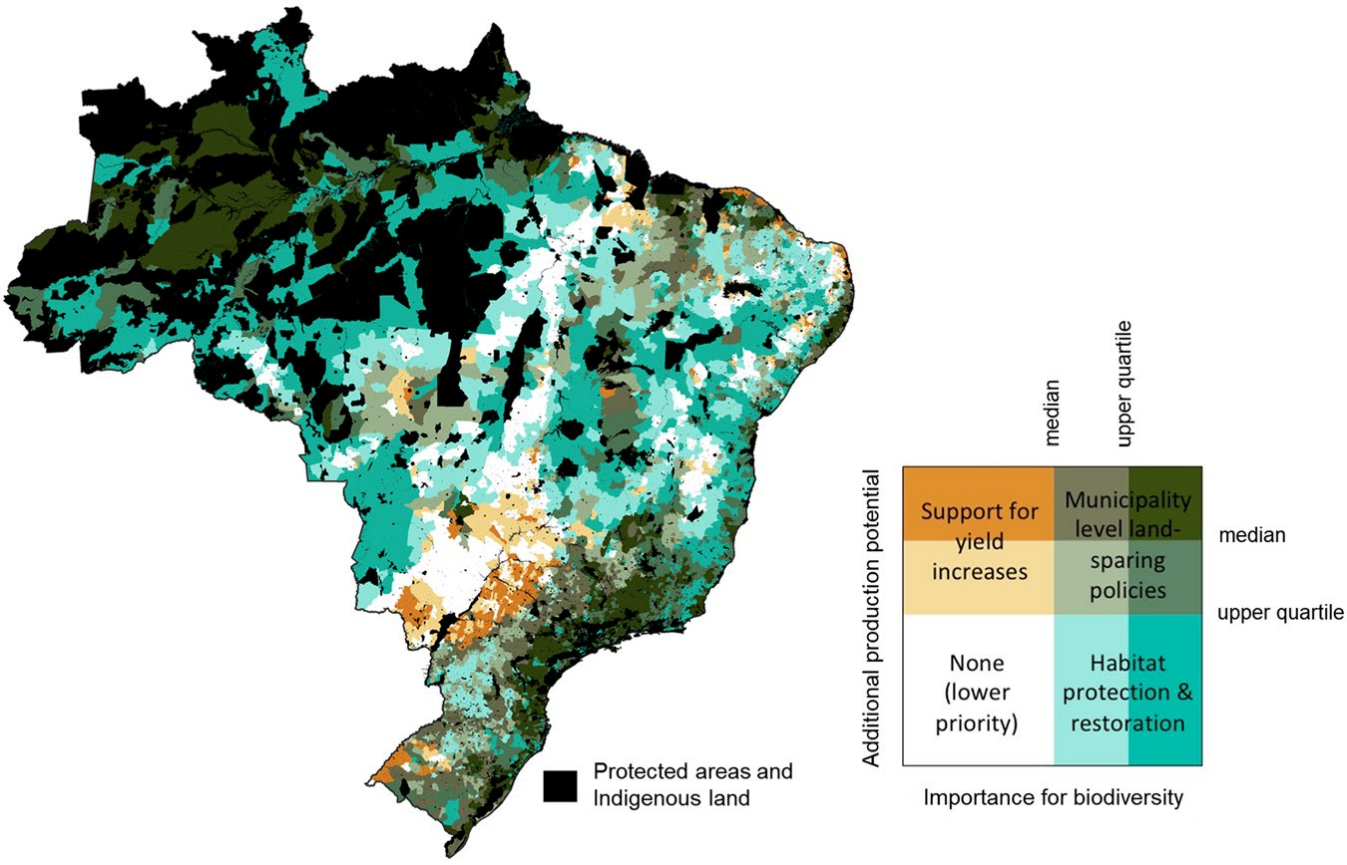
Map of broad policy categories appropriate for municipalities in Brazil. Colors are based on the relationships between additional production potential (per unit of agricultural area) and importance for biodiversity in each municipality. See Fig. 3 for graphical explanation of how these categories were defined. Black areas indicate protected areas, indigenous lands, and inland water bodies.

We next combined our agriculture and biodiversity layers by classifying municipalities into different categories according to their production potential and importance for biodiversity (Fig. 3). We repeated this classification using two different variants of each variable, and identified the highest category each municipality was assigned to (Fig. 4). We found rather few municipalities (567 of 5570) in the upper-left quadrant, where policies to “increase yields” without additional conservation measures might be appropriate. These are municipalities with high potential to produce additional food on existing agricultural land, but relatively low importance for either conservation or restoration. These municipalities host between 18% (contribution to domestic food supply) and 23% (total) of the country’s additional production potential, on 5% of its land area. They were concentrated particularly in the western parts of Paraná and São Paulo states, and the southern part of Mato Grosso do Sul.

There were also relatively few municipalities (596) in the lower-left (“lower priority”) quadrant, where all variants of the analysis found both below-median production potential and below-median importance for conservation and restoration. Just 6–7% of the country’s additional production potential was in these municipalities, covering 8% of the land area. A larger number of municipalities (1693) was found in the lower-right quadrant, where “habitat protection and restoration” policies would be appropriate. These are municipalities with above-median importance for either conservation or restoration, and below-median additional production potential. Municipalities in the lower right quadrant, and especially those of highest importance for biodiversity (blue-green in Fig. 4) were most prevalent in the Pantanal, the Amazon, and in parts of the Cerrado, Caatinga and Atlantic Forests. Although they made up almost half of the land area of Brazil (46%), only 12–16% of the country’s additional production potential was found in these municipalities.

The greatest number of municipalities (2714) was found in the upper-right quadrant, where “municipality-level land-sparing policies” are likely to be needed. These are municipalities with – in at least some variants of our analysis – above-median potential to produce additional food on existing agricultural land, and above-median importance for conservation or restoration. Municipalities with the highest production potential combined with highest importance for biodiversity (upper right part of upper-right quadrant; darkest green in Fig. 4) were concentrated in the Atlantic Forest of southeast and northeast Brazil, in the interior of the Amazon, with some such municipalities in the “arc of deforestation”, in the Matopiba region in northeast Brazil, and in other parts of the Cerrado. They made up 41% of the land area and supported 59–60% of the additional production potential.

**Figure 5:**
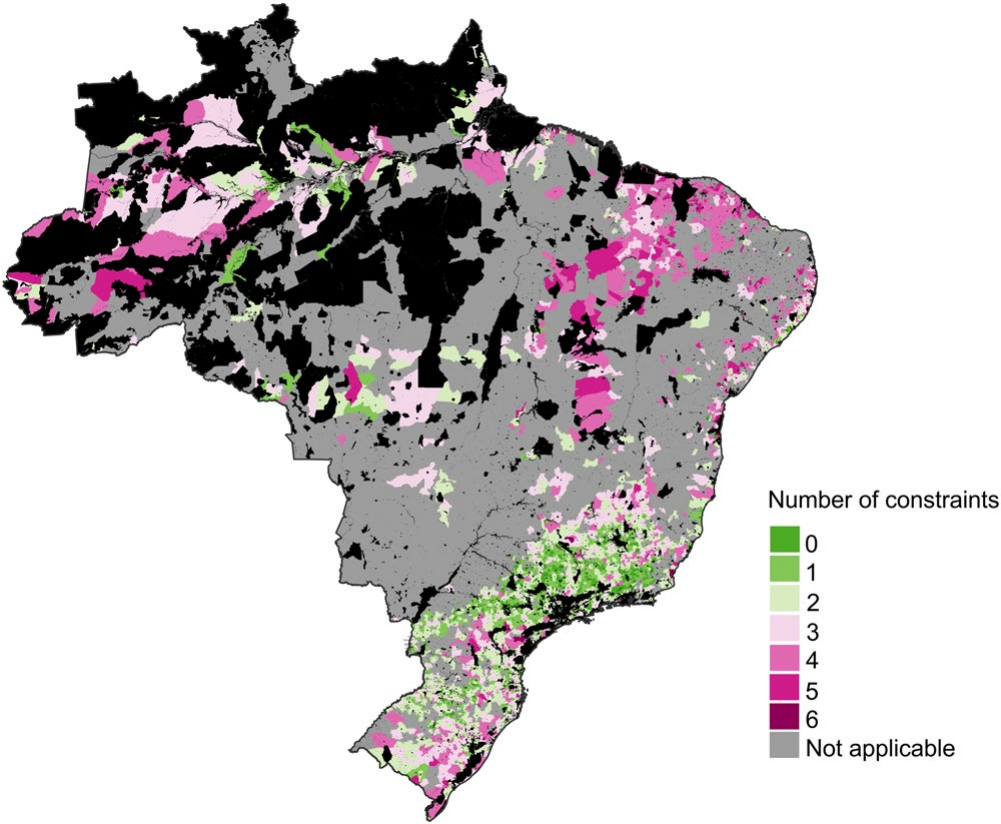
Map showing prevalence of six institutional constraints for land sparing, in municipalities identified as priorities for implementation of land-sparing policies. The constraints are defined and described in Table 1. Black areas refer to protected areas, indigenous lands, and inland water bodies.

We mapped a range of constraints for land sparing (Table 1) within those municipalities in the top right quadrant of Fig. 4, to identify areas where these constraints were most and least prevalent (Fig. 5). We identified some municipalities with few or no constraints. These occurred across most of Brazil, with a notable concentration in the Atlantic Forests and especially in southern Minas Gerais and parts of São Paulo. These municipalities (green in Fig. 5) are those where – at least when considering the set of constraints identified here – conditions are most favorable for the success of land-sparing policies. They are places where farmers have access to technical advice from extension agents; where there is intermediate labor availability (too much creates a stronger risk of leakage, while too little is a barrier to the adoption of more sustainable practices^33^); where more adults have completed school and thus might have greater agency; where land tenure is relatively secure; where there is relatively good compliance with the Forest Code; and where there is relatively little native vegetation available for legal clearance. Vegetation which is not available for legal clearance cannot be assumed to be safe, especially in the current political climate, but there is at least a clear legal pathway for conserving and restoring such areas. Within the 211 municipalities in the upper right sub-quadrant (darkest green in Fig. 4) and which had only one constraint, the most frequent constraint was labor (31%), followed by a surplus of native vegetation available for legal clearance (21%), insecure land tenure (18%) and lack of access to technical advice (18%). Municipalities with multiple constraints were concentrated in the Matopiba region, Brazil’s newest agricultural frontier (Fig. 5), suggesting that to spare land for nature in that region will be especially challenging.

## Discussion

By bringing together diverse, spatially explicit datasets, we present a flexible methodology for identifying where land-sparing policies are most needed, and where they are most likely to succeed. We focus on Brazil, a country of exceptional global importance for both biodiversity conservation and agriculture, but the methods used here could be adapted for any region with adequate data, and could be adapted to analyse other metrics such as carbon storage and emissions. Our illustrative analyses are a step towards a more fine-grained understanding of the potential for land-sparing policies in practice, and of the constraints they face. Our results underline the considerable technical potential for land sparing in Brazil, in line with other recent work, but also highlight the importance of understanding and addressing a range of social and economic constraints^2,3,34,35^.

Our analyses can help inform where to focus efforts to support land sparing in policy and practice. Yield gaps are widespread across the country, and widest in pasture. Increasing the efficiency of cattle production on pasture provides the greatest opportunities for reducing land demand. Despite this, closing yield gaps on pasture would make only a small addition to Brazil’s food supply^36^. Yield gaps are proportionally far smaller on cropland, and in many places, current yields are already close to their potential. However, cropland (even just the seven crops analyzed here) has the potential to supply more additional food than is possible through animal agriculture. Production of meat and milk is inefficient, but in consequence, presents the greatest biophysical potential to spare land for nature if the right policies, norms and incentives are in place.

What might those mechanisms look like? To promote land sparing, they should include instruments such as zoning, economic incentives or disincentives, targeted extension, and certification to link conservation and production outcomes^1^. Such linkages could be incorporated into agricultural extension, infrastructure planning, rural credit programs, farmer networks, and certification schemes^1,29^. Several real-world examples suggest the form these might take in Brazil. For example, a combination of stringent forest protection and clustered development of agribusiness in Mato Grosso has been linked to increases in stocking rates of cattle and double-cropping of soybeans with maize and cotton^35^. Various initiatives in the Brazilian Amazon have worked with cattle ranchers to increase pasture yields by 30–170% through use of rotational grazing, legumes, and other pasture management techniques, while at the same time improving compliance with the Forest Code^29^. Interestingly, all of the eight municipalities in which these initiatives occurred had below-median additional production potential, and above-median biodiversity importance, alongside the constraint of low labor availability, suggesting that strengthing environmental compliance should be a higher priority for these initiatives than supporting yield increases. More widely, through a combination of law enforcement, property registration, supply-chain commitments, protected area expansion and financial instruments, Amazon deforestation was reduced by ~70% in the decade from 2004, while beef and soybean production continued to increase^12,29,37^. The policies, norms and incentives to deliver land sparing are complex, but are becoming better-understood.

Our analysis points to the importance of finding the right scale at which to implement land sparing. We identified relatively few municipalities with both high potential to increase food production and low importance for biodiversity. More common were municipalities with low potential for increasing production and high biodiversity importance, or with high potential for both food production and biodiversity. This suggests that a strategy of focusing agricultural yield increases in some municipalities and conservation or restoration in others as part of a large-scale land sparing approach may not be sufficient. Instead, we suggest that conservation should be prioritized in some regions (such as the Pantanal), and municipality-level land-sparing policies developed in many others. In other words, coordinating both conservation and agricultural development within municipalities may be at least as important as large-scale segregation of conservation and farming.

We present a first attempt to identify and map some of the conditions that could help or hinder municipality-level land-sparing policies. These conditions, as well as conservation and agricultural needs, vary by municipality, calling for different strategies in different places. Areas where land-sparing initiatives are most likely to succeed are not necessarily those of highest production potential or conservation priority, but those where institutional constraints are minimal. In areas where constraints exist, efforts can be made to reduce their impact, through increasing access to technical advice, training the existing labor force, strengthening land tenure, monitoring and enforcing compliance with the Forest Code, and improving incentives to landowners for conservation measures such as private protected areas (Brazilian acronym: RPPNs). The data we synthesize here could be used in different ways by different actors to target such efforts to where they may be most successful.

Areas of high conservation importance are distributed across all of the country’s domains, from the Amazon to the Pampas, underlining the need for policies to extend beyond the forest domains that have hitherto received most attention^19^. The municipalities with the very highest levels of endemism and restoration priority were in the Atlantic Forest, as expected, but there were areas of exceptional importance elsewhere too, particularly in the Caatinga and Cerrado. Care is needed in interpreting these maps, as they are based on a limited set of taxa, emphasize endemism, and are influenced by biases in taxonomic and biogeographical knowledge^38^. However, we consider them a good starting point for identifying priorities, and an improvement over single-taxon maps of species richness.

The inherent inefficiency of livestock production also points to the value of demand-side measures, to reduce consumption of meat and milk and to shift diets towards plant-based alternatives^39^. On the supply side, increasing livestock productivity would do little to increase the food supply, but it could help to slow or reverse the expansion of agricultural area in Brazil if combined with “land-neutral agricultural expansion”^38^, adequate protection of native vegetation, and forest restoration^40,41^. Using methods such as integrating silviculture into livestock systems to restore degraded pastures (at least half of Amazon and Cerrado pastures^42^) could help to minimize the negative environmental effects of increasing yields. There is much interest in integrated crop–livestock–forestry systems in Brazil, but as yet relatively limited evidence for their productive and environmental benefits^43^. Incentives to increase crop and livestock yields are just one of the steps needed to spare land for nature^26^, and care must be taken not to encourage agricultural expansion through a rebound or backfire effect^6^.

Increasing the yields of export commodities such as soybeans and sugarcane is less likely to contribute towards the objective of sparing land for nature than increasing yields of domestically-consumed staple crops such as rice and wheat. Some technologies are less compatible with land sparing than others. For example, more efficient deforestation techniques^44^ will promote deforestation, while investments in existing farmed land (such as installing drip irrigation systems) are more likely to promote the consolidation of agricultural production on existing farmland. Limited labour availability could reduce the risk of leakage, but could also make it more difficult or expensive for farmers to adopt improved agricultural practices^33^.

Our datasets and analyses have various limitations. Large-scale agricultural and biodiversity datasets do not fully reflect local conditions. Our maps of biodiversity are only as complete as existing knowledge of species and their distributions, and other environmental outcomes, such as biomass, could also merit consideration. Municipalities are treated as units, but even within municipalities, there may be great variation in soil type, terrain and other variables. We averaged estimates of future production potential across multiple climate models, but only one trajectory will come to pass. Energy ouptut is an imperfect metric of food production, and our metric of contribution to domestic food supply goes only partway to taking account of beneficiaries and end-uses. As described in the Introduction, there is some evidence for the influence of the variables shown in Table 1 on land-use outcomes, but more is needed, and cut-offs (such as median values) for mapping them as constraints are arbitrary. Other variables such as poverty, population density, and government investment in social protection programs may also be important. To inform decision-making, all such variables will need to be considered in the context of local particularities, alongside the expected marginal costs and benefits of different conservation actions.

These limitations notwithstanding, our results indicate some promising ways forward for reconciling biodiversity conservation and agricultural production in Brazil, including reducing demand for meat and milk, restoring degraded pastures using methods such as integrated crop–livestock–forestry systems, shrinking the land area used for livestock, replacing some pasture with cropland, and strengthening efforts to protect and restore native vegetation. The results also help to identify where such efforts might be most effective, as well highlighting constraints in other parts of Brazil that will need to be overcome for land sparing to succeed. Our data and analyses can be adapted to inform conservation planning, assist NGO engagement with the agricultural sector, aid corporate sustainability efforts, and to develop policies, norms and incentives to link conservation and production outcomes within municipalities. With such efforts, Brazil could build on its existing achievements to become a world leader in reconciling biodiversity conservation and agricultural production.

## Methods

### Study scope

Our analysis included all of the land area of Brazil. We collated data at the scale of municipalities. For datasets in raster format, we first mapped agricultural potential and priorities for conservation at a 10 km or 1 km resolution. Then we summarized the results at a municipality scale. We converted all yield estimates from units of mass (tonnes/ha) to units of food energy (gigajoules or petajoules per hectare, GJ/ha or PJ/ha) using standard conversion factors (Supplementary Table 3). For further details of methods, see the Supplementary Materials.

### Current agricultural production and yields: crops

We obtained estimates of current production and cultivated area of seven major crops (rice, sugarcane, cassava, maize, soybean, sorghum and wheat) at municipality level (see Supplementary Files for data sources). To estimate yields, we divided production by area.

### Current agricultural production and yields: cattle

We estimated beef production per municipality using information on herd sizes combined with region-level statistics on slaughter rates and mean carcass weights. We obtained estimates of current milk production (in liters) per municipality from IBGE. These were converted to tonnes using a conversion factor of 1.032 kg/L. To estimate yields, we divided production by cultivated pasture area, estimated for each municipality by LAPIG (https://www.lapig.iesa.ufg.br/lapig/). We also calculated the number of heads per hectare, and converted this to animal units (AU) per hectare, for comparison with the potential yields. To convert to animal units, we used state-level conversion factors (AU per head) calculated by RFB from a multiple regression based on detailed analysis of herd composition in each state (Supplementary Table 2).

### Additional production potential: crops

The yield gap is defined as the difference between observed crop yields and those attainable (potential yields) in a given region. Additional production potential refers to how much more of a product could be produced by closing yield gaps in a municipality. We obtained maps of yield gaps for crops from the EarthStat website. These raster maps were developed by Mueller *et al*.^30^, and show yield gaps at a five arc-minute (~10 × 10 km) scale, with reference to the yields attained in areas with similar climate. We calculated the average yield gap in each Brazilian municipality for the seven focal crops. Then, we calculated the additional production potential for each crop in each municipality by multiplying the yield gap by the area of each crop, using data on cultivated area in hectares from IBGE.

### Additional production potential: cattle

To estimate yield gaps for beef and milk production, we used projections of the potential production of pasture grasses under different climate scenarios for the 2020s from the Global Agro-Ecological Zones (GAEZ) data portal. GAEZ estimates the production potential for pasture, considering the agronomically possible upper limit of yield, and agro-climatic, soil, and terrain conditions for a specific level of agricultural inputs and management conditions^45^.

We calculated the mean of all 11 projections for high-input, rainfed pasture grasses to get a single raster map with a mean projected value for each pixel. These projections were based on 11 variants of four major climate models (CCCma CGCM2 A2 and B2; CSIRO Mk2 A2, B1 and B2; Hadley CM3 A1FI, A2, B1 and B2; and MPI ECHAM4 A2 and B2). We excluded additional projections referring to specific subsets of grasses (C3 or C4). The map units were 10 kg dw/ha per year, and thus, were converted to yields in kg dw/ha by multiplying all values by 10. We converted the potential grass yields to potential stocking rate in animal units (AU/ha), assuming a daily feed intake of 8 kg/AU/d and grazing efficiency of 50%^3^. We then calculated the difference between current and potential stocking rates, and converted the result to beef and milk equivalents using the region-specific conversion factors already established.

### Contribution to domestic food supply

As an estimate of the potential contribution of closing yield gaps to domestic food supply, we calculated the proportion of each crop or livestock product that is consumed by people in Brazil. We subtracted net exports, waste and non-food uses from total production, and for crops used as feed, we used estimates of feed conversion efficiency to convert their energy value to estimates of their food value to people. Our metric is imperfect, because the contribution of crops and livestock to food security also depends on what form the food is consumed in, and by whom, but it serves as an indication of the contribution that closing yield gaps could make to feeding people in Brazil.

We used estimates of production, imports, exports and allocation from FAOSTAT for the most recent year with complete data (2013) to calculate the proportion of each crop and livestock product that contributes to food supply in Brazil (see Supplementary Files). We then multiplied these proportion values by the additional potential production for each product.

### Conservation and restoration priority

To identify those municipalities where the protection and restoration of native vegetation is most needed, we used a metric of the importance of each grid cell within each municipality for vertebrate species. This metric is similar to the “impact score” developed by Buchanan *et al*.^46^. To calculate it, we estimated the proportion of each species’ global range that occurs in each grid cell, and then, for each cell, summed those proportions for all species in the cell. This gave us a metric of the biodiversity importance (BI) of each cell.

We used global species distribution maps for birds, mammals and amphibians from IUCN and BirdLife International. We selected all species that occur in Brazil which are indicated as “extant”, “probably extant”,”native”, “reintroduced”, or “origin uncertain”, and converted their polygon distribution maps into rasters of 1 km × 1 km spatial resolution. The importance of each cell for each species was calculated as the proportion of its global range in that cell. For example, if a species occurs in 100 cells, the importance of each cell, for that species, will be 0.01.

We then combined the BI metric with a map showing remaining native vegetation. To identify areas of greatest importance for conserving native vegetation, we multiplied BI by the proportion of remaining native vegetation in each 1-km raster cell. To identify areas of greatest importance for restoration, we multiplied BI by the proportion of land that had been converted to uses other than native vegetation or urban. Both importance for conservation and for restoration were averaged across all grid cells in each municipality. For more details on our map of native vegetation, see Supplementary Methods.

### Classification of municipalities

To provide a broad assessment of which sorts of interventions and policies might be most useful in different parts of Brazil, we classified municipalities into nine categories, within four quadrants. The quadrants were defined according to both their production potential and their importance for biodiversity, and delineated by the median values of each variable (see Fig. 3). The quadrants were further subdivided at the upper quartile, resulting in a total of nine categories. So that our conclusions were not overly sensitive to one set of assumptions, we repeated this division for two variants of each of the production and biodiversity variables, resulting in four combinations of variables. For production, we used additional production potential and additional production potential adjusted to reflect contribution to domestic food supply. For biodiversity, we used importance for conservation and importance for restoration. We identified the highest category each municipality was assigned to in any of the four combinations (Fig. 4).

Municipalities with high potential to produce additional food on existing agricultural land, but relatively low importance for biodiversity, are those where it might be best to focus support to farmers and ranchers to increase their yields. Municipalities with high importance for biodiversity, but low agricultural potential, are places where efforts to protect and restore habitats might be most straightforward. Municipalities where both biodiversity importance and additional production potential are high are those where there is a need for municipality-level land-sparing policies. These are the places where conservation might be most complicated and expensive, and where efforts to stimulate agriculture might be most dangerous.

### Modifying conditions for land sparing

We assembled data on social and economic variables expected to influence the success of land sparing policies. The variables were selected based on the closest match we could find between available data and the variables expected to be important^5^. They are described, together with their expected influence on the success of land-sparing policies, in Table 1.

### Mapping constraints for land-sparing policies

To begin to understand where different factors might impede or facilitate the success of land-sparing policies, we mapped the distribution of five key constraints across municipalities identified as priorities for land-sparing policies. These constraints are defined in Table 1.

## Supplementary information

Supplementary Information

Supplementary Information2.

## Data Availability

Data made available as Supplementary Online Files.
