# Supplementary material for: Characterising the spatial distribution of opportunities and constraints for land sparing in Brazil
Source: Sci Rep. 2020 Feb 6;10:1946. doi: 10.1038/s41598-020-58770-5 (PMC7005321; doi:10.1038/s41598-020-58770-5)
Supplement: Supplementary file 1 — Supplementary Information [file 41598_2020_58770_MOESM1_ESM.docx]

Supplementary information for

**Characterising the spatial distribution of opportunities and constraints for land sparing in Brazil**

Juliana Silveira dos Santos,^1,2^*, Rafael Feltran-Barbieri^1,3^ , Ellen S. Fonte^1^, Andrew Balmford^4^, Veronica Maioli^1^, [Agnieszka Latawiec](https://www.google.com.br/search?client=firefox-b-ab&dcr=0&q=agnieszka+latawiec&spell=1&sa=X&ved=0ahUKEwj3lcD4qIDaAhWTPpAKHUCsBEcQkeECCCUoAA)^1,5,6,7^, Bernardo B. N. Strassburg^1,5,8,9^, Benjamin T. Phalan^1,10^*

* Corresponding authors: juliana.silveiradossantos@gmail.com; bphalan@ufba.br


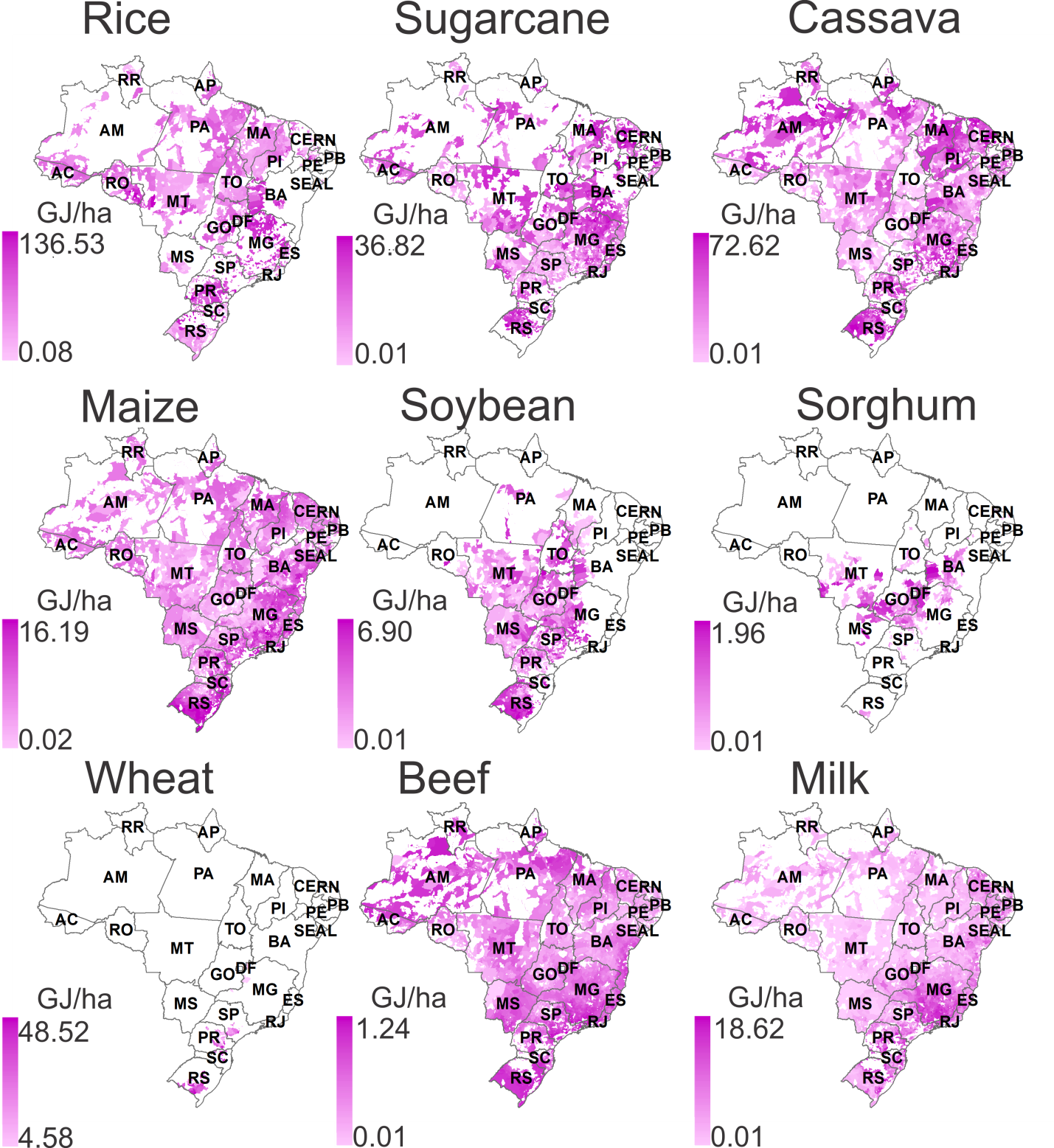


**Supplementary Figure 1.**  **Additional production potential is expressed as the additional food energy that could be produced per hectare of each cropland and pasture in each municipality.** These estimates are adjusted to reflect contributions to domestic food supply (excluding non-food uses, net exports and waste) as described in the text. White areas correspond to protected areas, indigenous land and municipalities with zero additional potential.


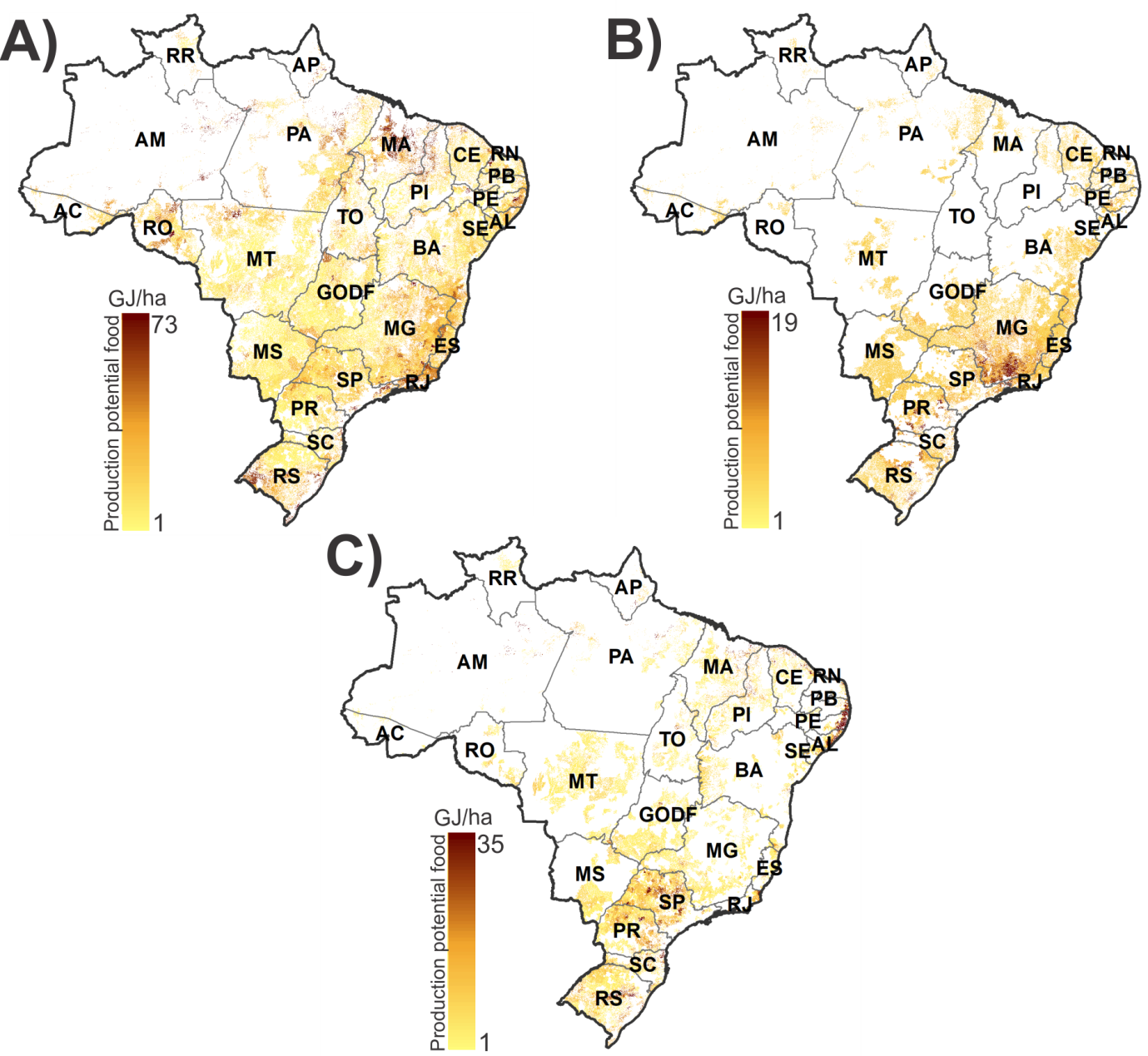


**Supplementary Figure 2.** **Map of municipalities in Brazil showing additional production potential on current farmland of (A) seven major crops (rice, sugarcane, cassava, maize, soybean, sorghum and wheat), (B) beef and milk and (C) crops, beef and milk combined.** Additional production potential is expressed as the additional food energy that could be produced per hectare of all farmland (croplands and pasture) in each municipality. These estimates are adjusted to reflect contributions to domestic food supply (excluding non-food uses, net exports and waste) as described in the text. A) crops; B) beef and milk and C) crops plus beef and milk. White areas correspond to protected areas, indigenous land and municipalities with zero additional potential.


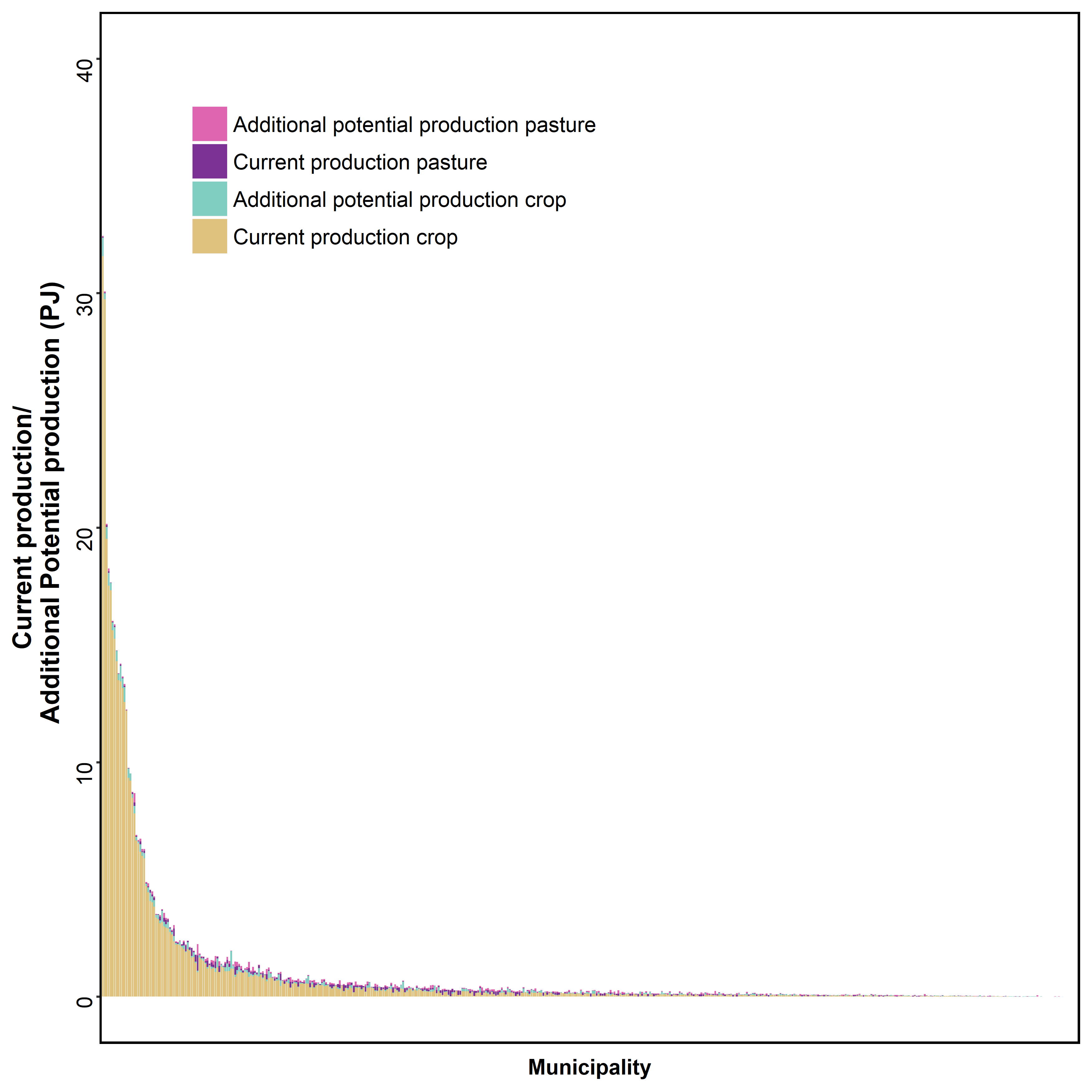


**Supplementary Figure 3.** **Current production and additional production potential, in PJ of food energy, for seven major crops (cassava, maize, rice, sorghum, soybean, sugarcane and wheat) on existing cropland and beef and milk on existing pasture.** Each bar represents one municipality, ordered by current production. Data are shown for the 496 municipalities of the Amazon.


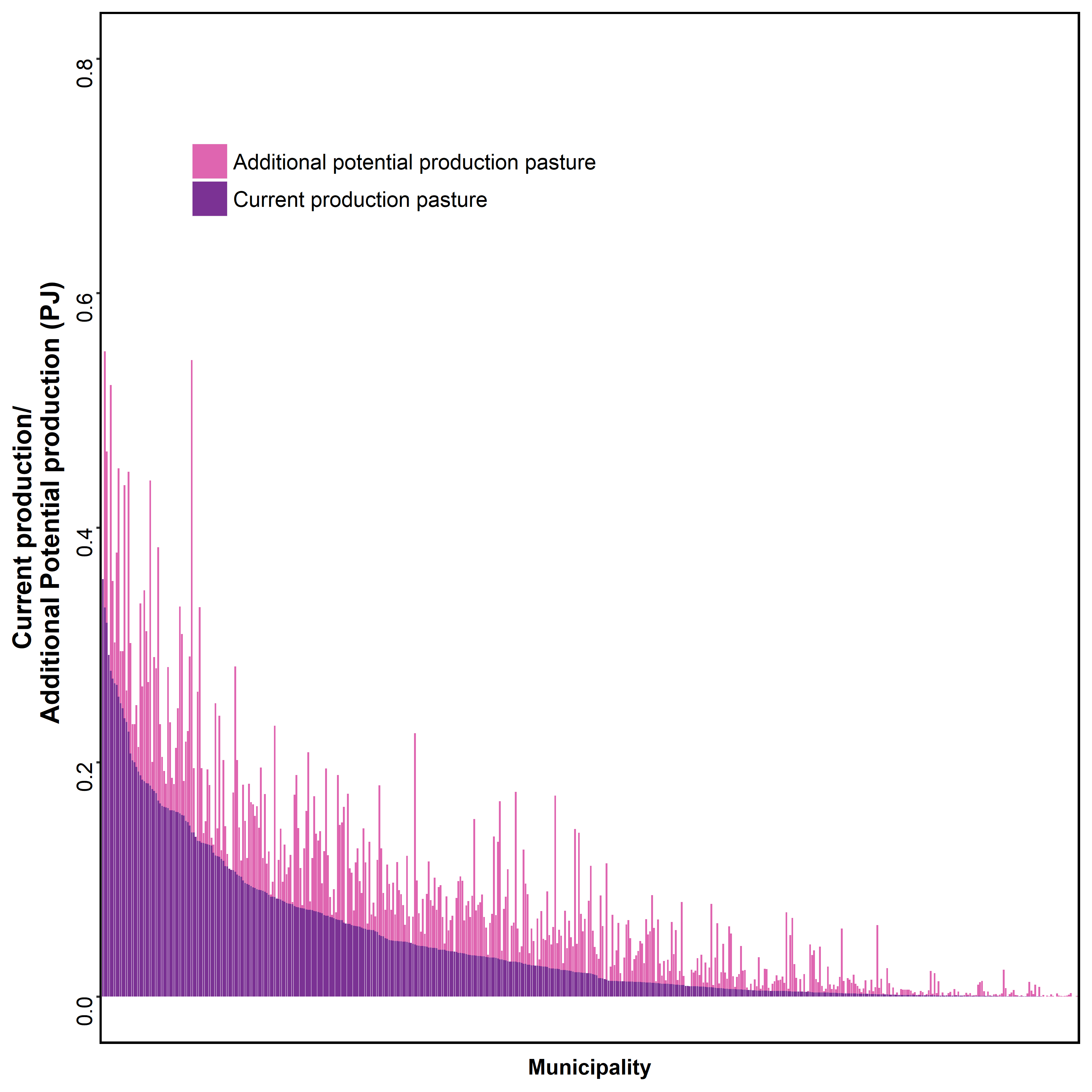


**Supplementary Figure 4.** **Current production and additional production potential, in PJ of food energy, of beef and milk on existing pasturelands.** Each bar represents one municipality, ordered by current production. Data are shown for the 496 municipalities of the Amazon.


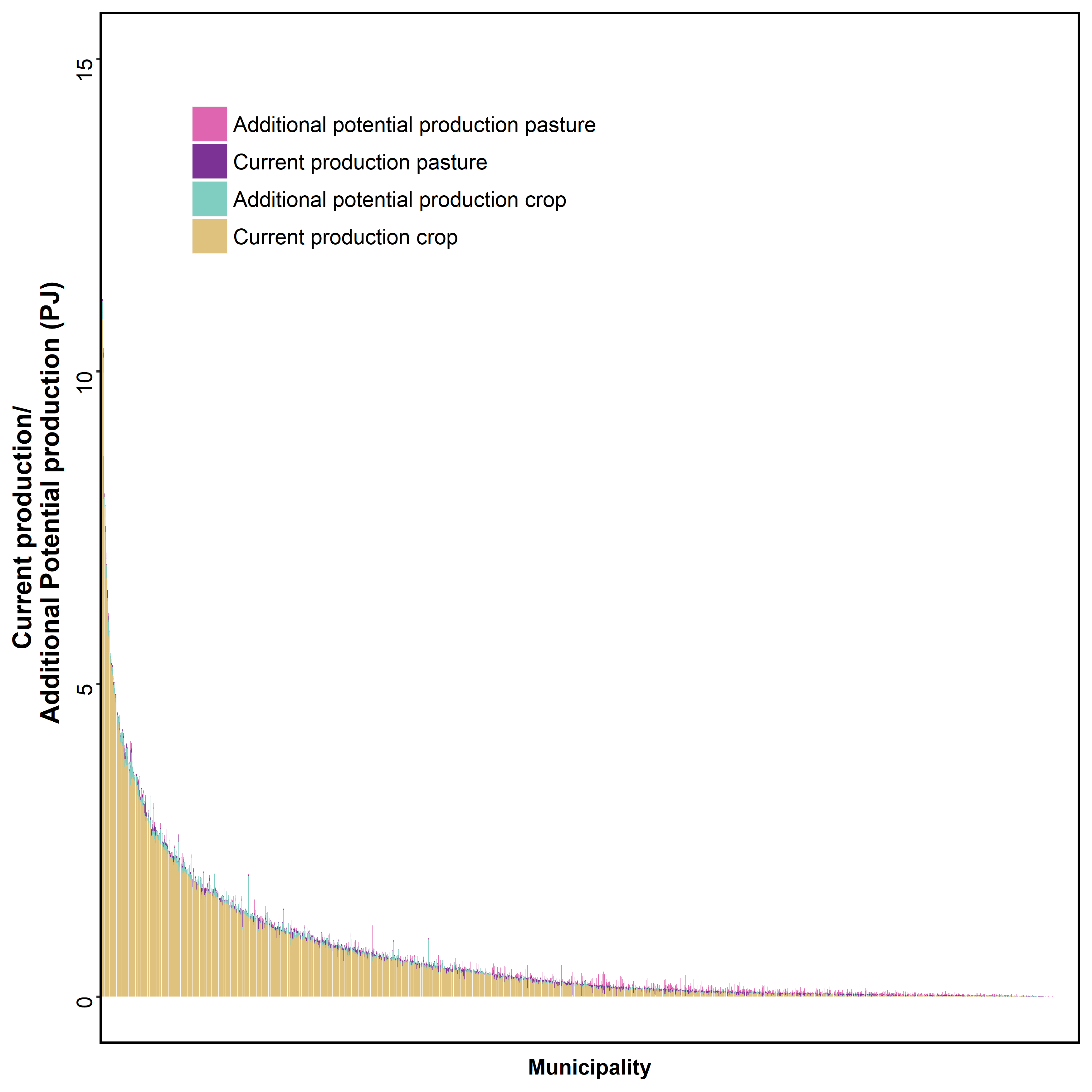


**Supplementary Figure 5.**  **Current production and additional production potential, in PJ of food energy, for seven major crops (cassava, maize, rice, sorghum, soybean, sugarcane and wheat) on existing cropland and beef and milk on existing pasture**. Each bar represents one municipality, ordered by current production. Data are shown for the 2729 municipalities of the Atlantic Forest.


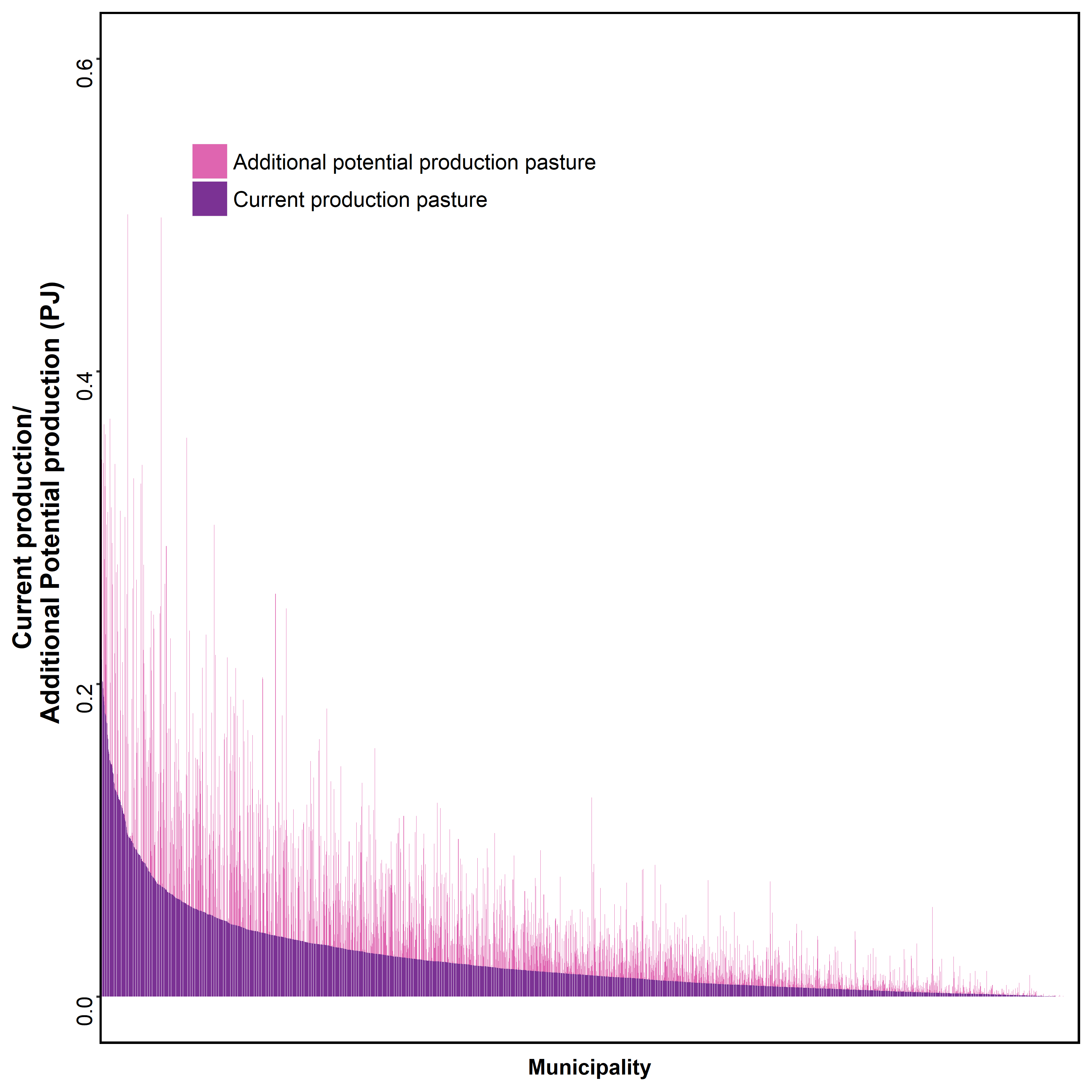


**Supplementary Figure 6.** **Current production and additional production potential, in PJ of food energy, of beef and milk on existing pasturelands.** Each bar represents one municipality, ordered by current production. Data are shown for the 2729 municipalities of the Atlantic Forest.


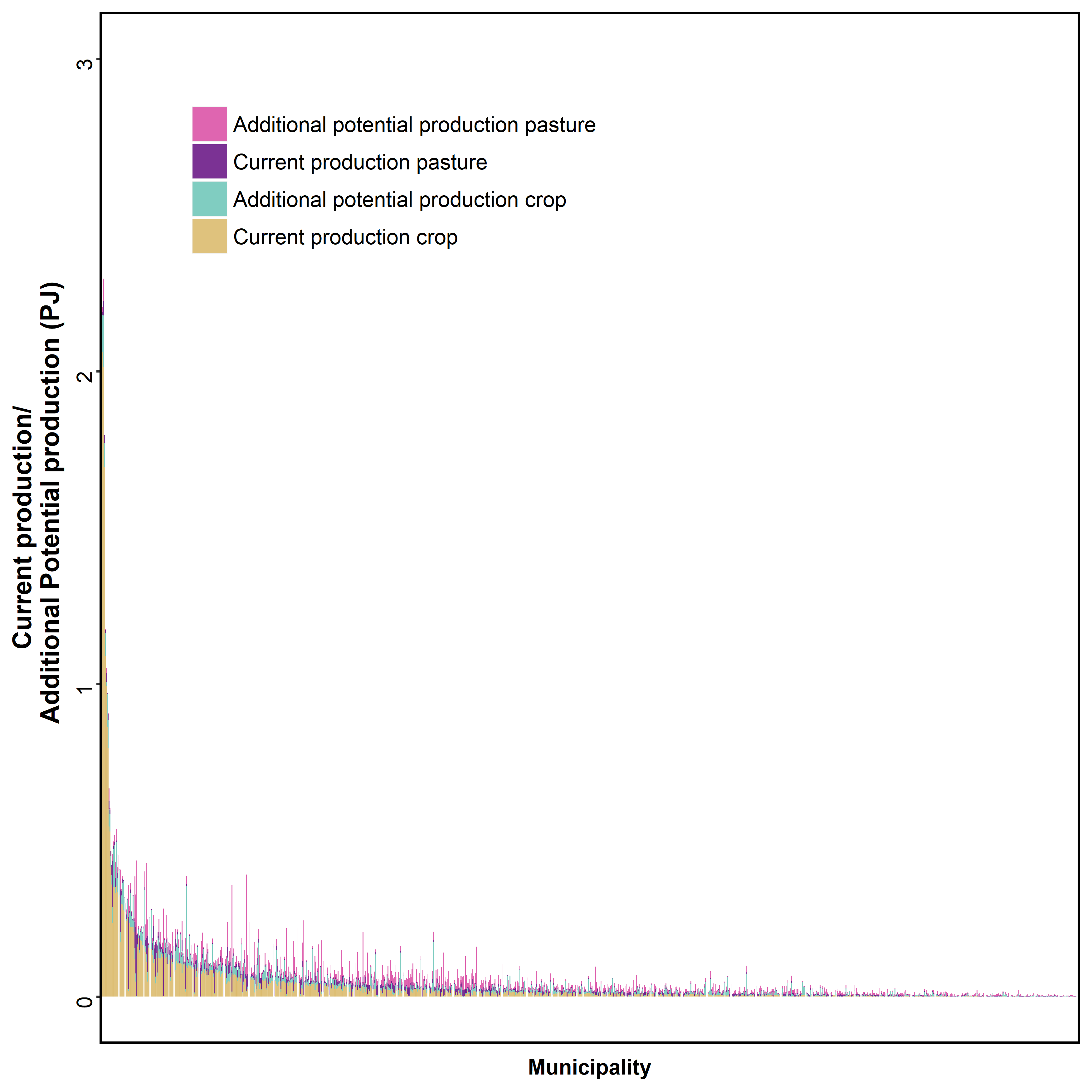


**Supplementary Figure 7. Current production and additional production potential, in PJ of food energy, for seven major crops (cassava, maize, rice, sorghum, soybean, sugarcane and wheat) on existing cropland and beef and milk on existing pasture.** Each bar represents one municipality, ordered by current production. Data are shown for the 1100 municipalities of the Caatinga.


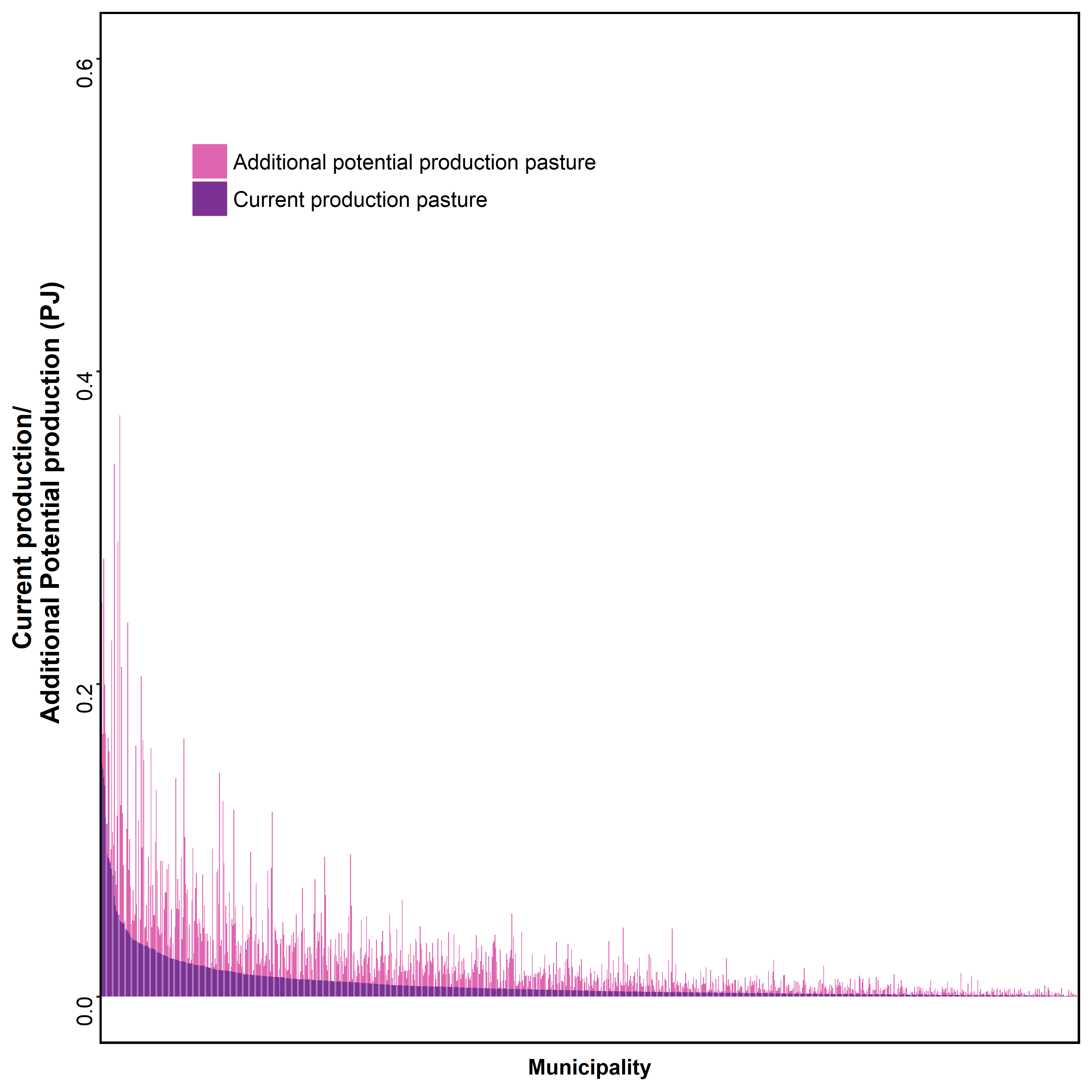


**Supplementary Figure 8. Current production and additional production potential, in PJ of food energy, of beef and milk on existing pasturelands.** Each bar represents one municipality, ordered by current production. Data are shown for the 1100 municipalities of the Caatinga.


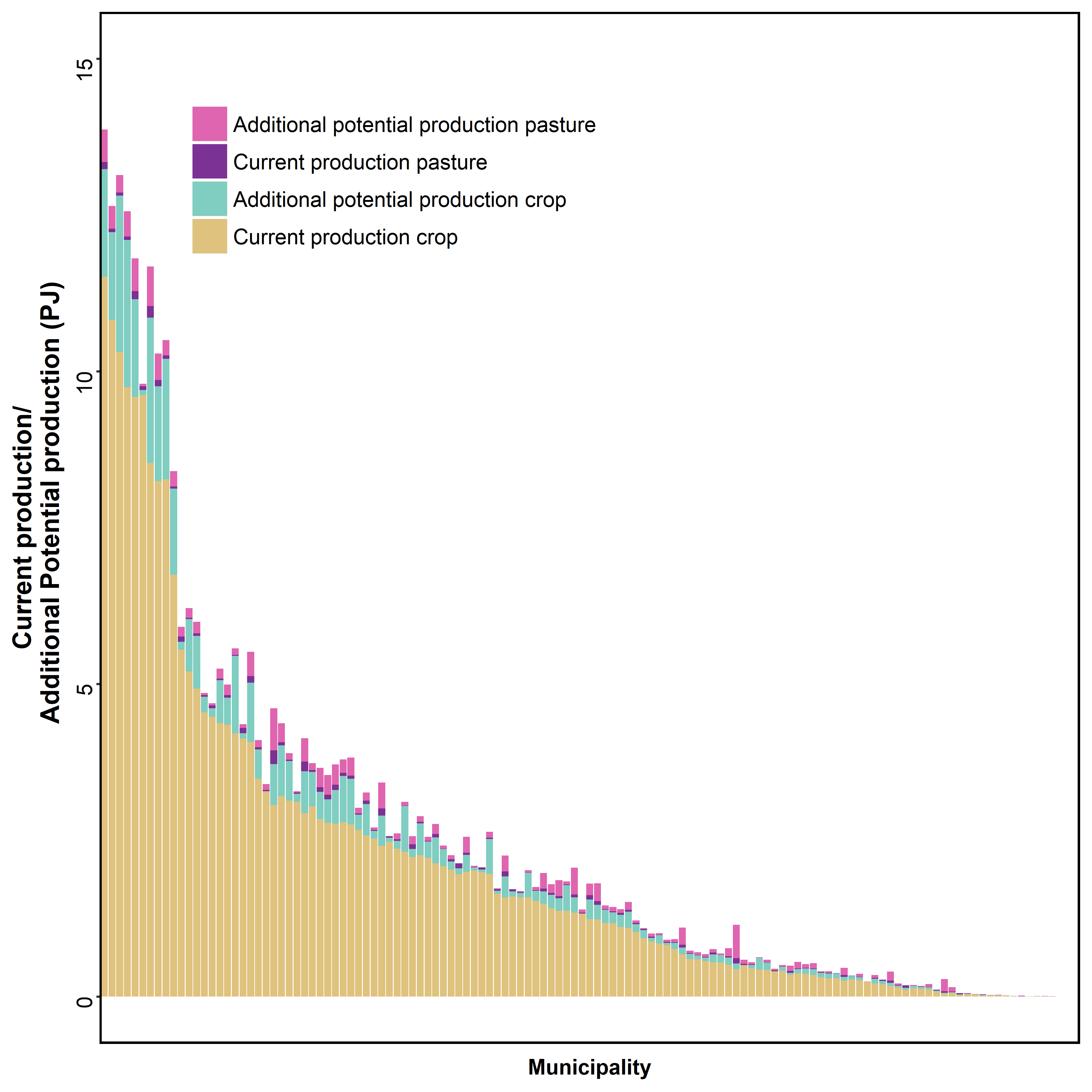


**Supplementary Figure 9.** **Current production and additional production potential, in PJ of food energy, for seven major crops (cassava, maize, rice, sorghum, soybean, sugarcane and wheat) on existing cropland and beef and milk on existing pasture.** Each bar represents one municipality, ordered by current production. Data are shown for the 127 municipalities of the Pampa.


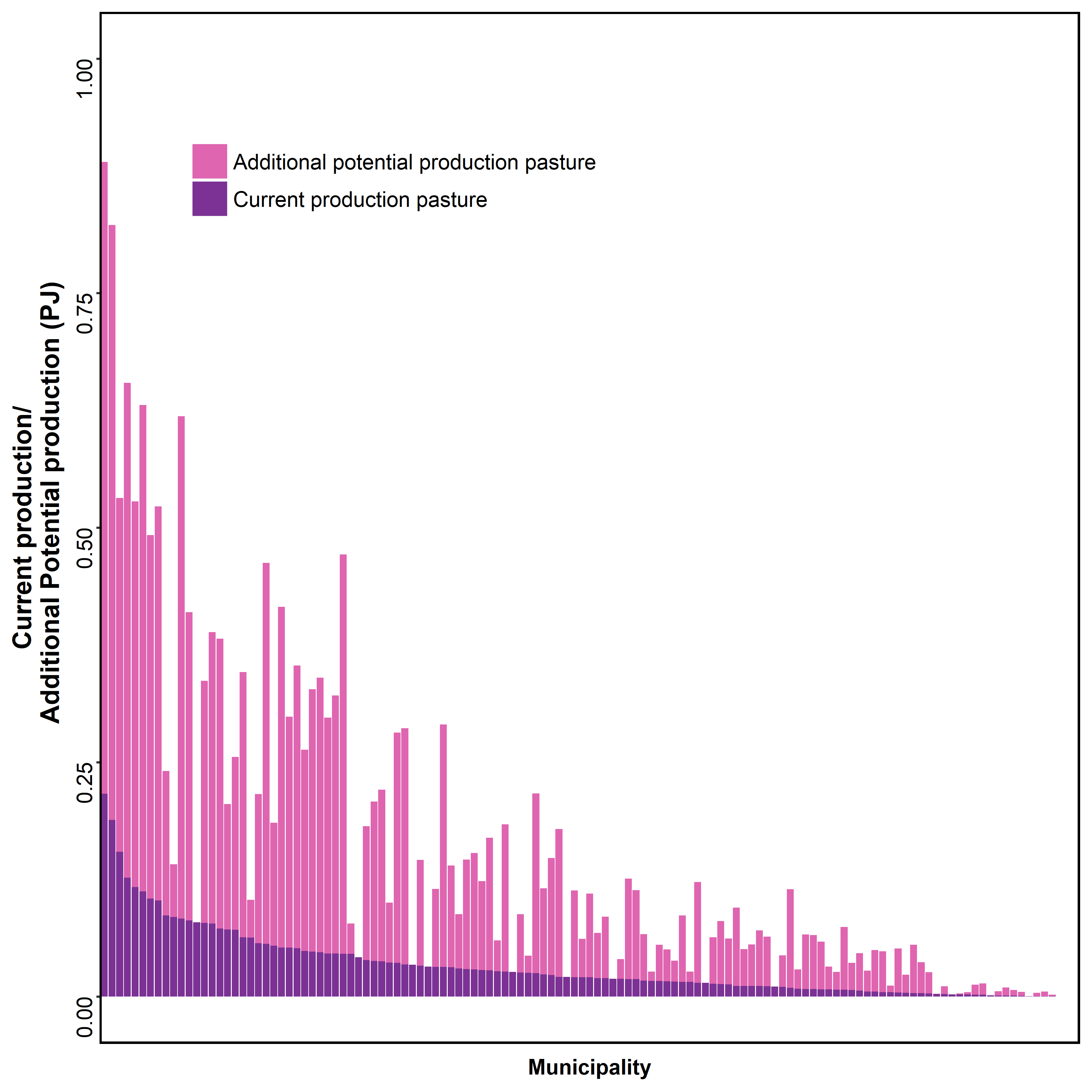


**Supplementary Figure 10.** **Current production and additional production potential, in PJ of food energy, of beef and milk on existing pasturelands.** Each bar represents one municipality, ordered by current production. Data are shown for the 127 municipalities of the Pampa.


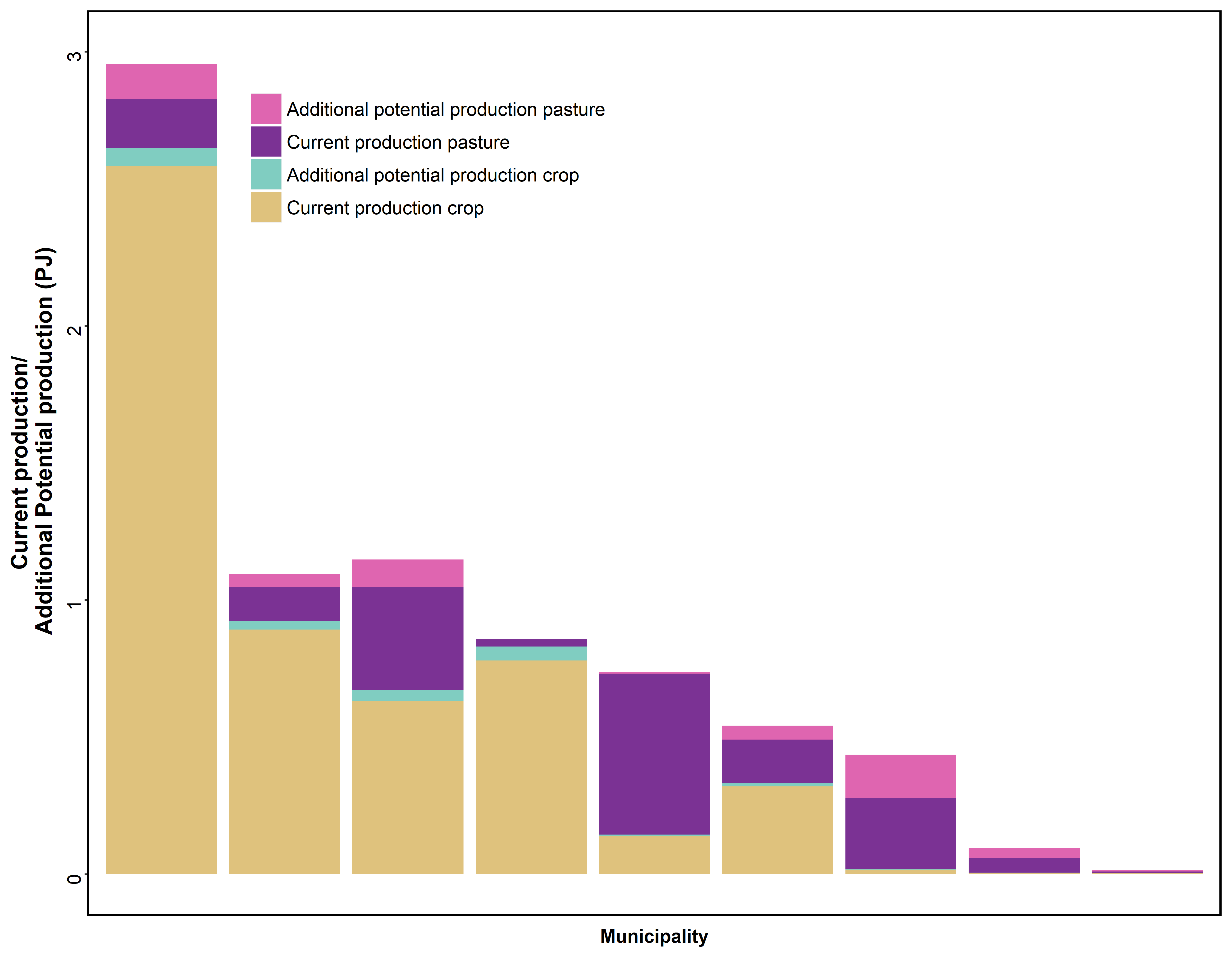


**Supplementary Figure 11.** **Current production and additional production potential, in PJ of food energy, for seven major crops (cassava, maize, rice, sorghum, soybean, sugarcane and wheat) on existing cropland and beef and milk on existing pasture.** Each bar represents one municipality, ordered by current production. Data are shown for the nine municipalities of the Pantanal.


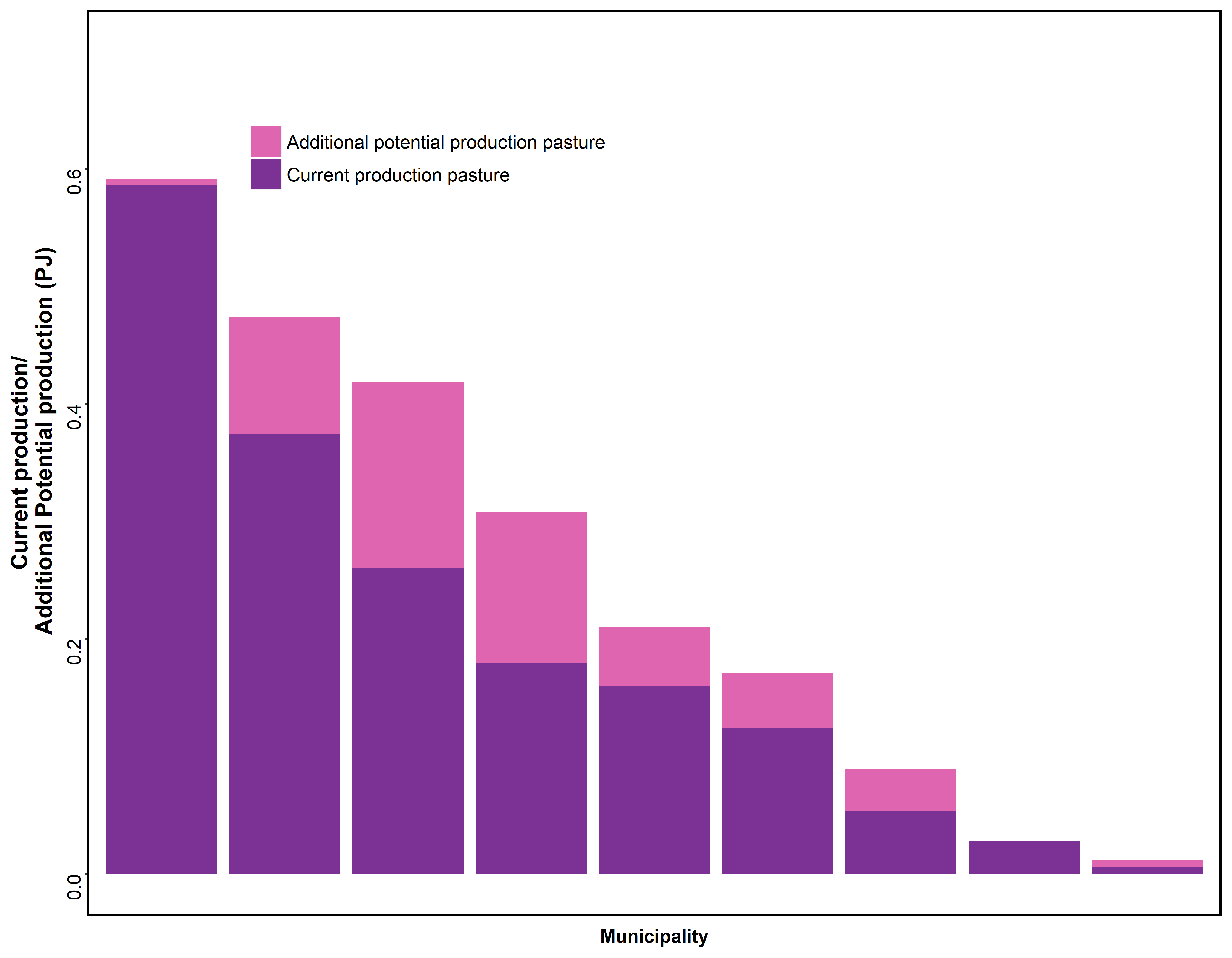


**Supplementary Figure 12. Current production and additional production potential, in PJ of food energy, of beef and milk on existing pasturelands.** Each bar represents one municipality, ordered by current production. Data are shown for the nine municipalities of the Pantanal.


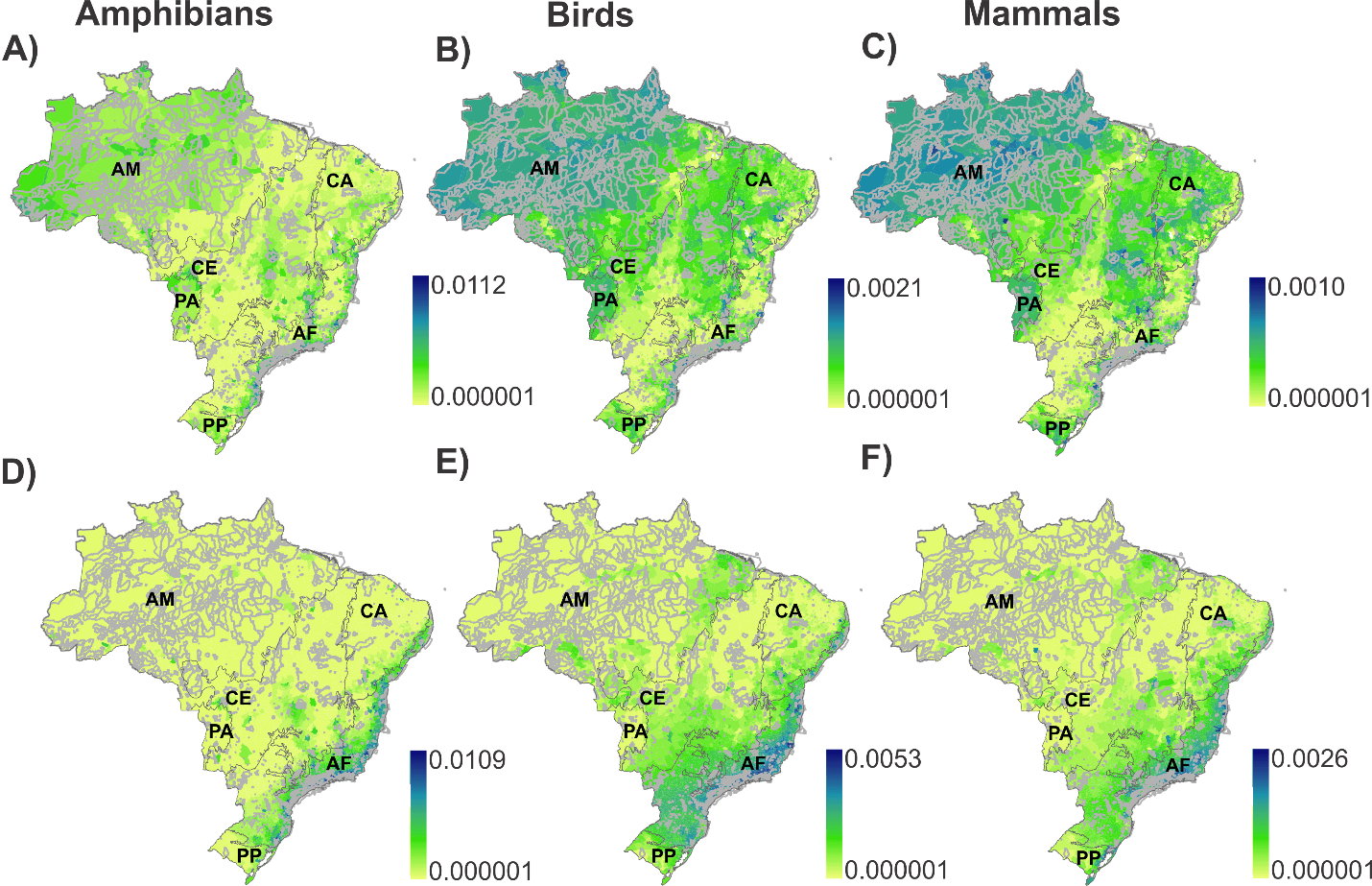


**Supplementary Figure 13. Maps of the importance for conservation (A-C), and importance for restoration (D-F) for amphibians, birds and mammals.** Polygons outlined in grey indicate Protected areas and Indigenous Land. Conservation importance is calculated as the proportion of remaining natural vegetation in each municipality, multiplied by the mean biodiversity importance (1 km resolution), while restoration priority is the proportion of cleared natural vegetation (excluding urban areas) multiplied by the mean biodiversity importance. The white areas correspond to zero importance areas. Abbreviations refer to domains (AM: Amazon, PA: Pantanal, CE: Cerrado, CA: Caatinga, AF: Atlantic Forest and PP: Pampa).

**Calculation of current production for beef and milk per municipality**

Estimates of beef production were available only at state level. To estimate production at municipality level we used information on herd sizes combined with region-level statistics on slaughter rates and mean carcass weights (**Eq.1**).

${CBP}_{t}=B_{TH}*{CH}_{m}$ **Eq.1**

Where: CBP_t_ is the current beef production per municipality in tonnes; B_TH_ is the total cattle carcass weight produced in one year divided by the total number of cattle heads in the region (not just those slaughtered) in tonnes and CH_m_ is the total number of cattle heads per municipality.

We obtained the total number of cattle heads per municipality from the IBGE’s Livestock Research per municipality (in Portuguese “Pesquisa Pecuária Municipal”) in the year 2015 (<https://sidra.ibge.gov.br/tabela/74> ). We calculated the carcass weight per total heads (not just those slaughtered) for each region, using carcass yield data from: <https://www.scotconsultoria.com.br/noticias/artigos/37616/rendimento-de-carcaca-em-frigorificos-do-brasil-.htm>

We used this conversion factor for each municipality in that region (Supplementary Table 1). Region-level rates were used because at state level there are anomalies, most notably that Distrito Federal (DF) appears to slaughter many more cattle than would be expected if they all came from within the Distrito Federal. Regions were as in Supplementary Table 1. However, there are substantial regional differences, so regional-level rates were considered preferable to a national average.

**Supplementary Table 1.** Conversion factors from total heads (not just those slaughtered) to tonnes of beef, by major region, based on carcass yield. The regions are composed of different States, according to an official classification. However, we also separated an unofficial region called Matopiba that corresponds to a new Brazilian agricultural frontier.

| **Region** | **Beef per total heads (tonne)**  **[Total carcass weight / total heads]** |
| --- | --- |
| Amazon: AC, AM, AP, PA, RO, RR | 0.032047 |
| Matopiba: MA, TO, PI, BA | 0.027860 |
| Northeast: AL, CE, PB, PE, RN, SE | 0.020574 |
| Southeast: SP, ES, MG, RJ | 0.041178 |
| South: PR, SC, RS | 0.028953 |
| Centre-west: DF, GO, MS, MT | 0.038648 |

Where: Amazon region corresponds to states: Acre (AC), Amazonas (AM), Amapá (AP), Pará (PA), Rondônia (RO) and Roraima (RR); Matopiba corresponds to states: Maranhão (MA), Tocantins (TO), Piauí (PI) and Bahia (BA); Northeast corresponds to states: Alagoas (AL), Ceará (CE), Paraíba (PB), Pernambuco (PE), Rio Grande do Norte (RN) and Sergipe (SE); Southeast corresponds to states: São Paulo (SP), Espírito Santo (ES), Minas Gerais (MG) and Rio de Janeiro (RJ); and South corresponds to states: Paraná (PR), Santa Catarina (SC) and Rio Grande do Sul (RS).

We obtained estimates of current milk production in litres per municipality from the IBGE’s Livestock Research of the year 2015 (<https://sidra.ibge.gov.br/Tabela/3939> ). We converted from thousands of litres of milk to tonnes, using a conversion factor from Embrapa: <http://www.agencia.cnptia.embrapa.br/Agencia8/AG01/arvore/AG01_196_21720039246.html> (**Eq.2**).

${CMP}_{t}={CMP}_{l}*CF$ **Eq.2**

Where: CMP_t_ is the current milk production per municipality in tonnes; CMP_l_ is the current milk production in liters and CF is the Embrapa’s conversion factor to convert one thousand litres of milk to tonnes. The CF value is 0.001032.

**Supplementary Table 2.** Conversion factors from livestock animal units (AU) of cattle (standing stock) to tonnes of milk produced per year in each state of Brazil, including conversion factor from heads to AU.

| **State** | **Heads of cattle** | **Milk production thousand litres** | **AU/head average** | **Thousand litres milk per AU** | **Tonnes milk per AU** |
| --- | --- | --- | --- | --- | --- |
| Acre | 2,799,673 | 49,009 | 0.71257 | 0.02457 | 0.02533 |
| Alagoas | 1,253,121 | 305,060 | 0.72604 | 0.33530 | 0.34569 |
| Amapá | 167,529 | 20,867 | 0.76606 | 0.16260 | 0.16764 |
| Amazonas | 1,405,208 | 75,556 | 0.68560 | 0.07843 | 0.08086 |
| Bahia | 10,824,134 | 1,276,108 | 0.78453 | 0.15027 | 0.15493 |
| Ceará | 2,597,139 | 580,691 | 0.65451 | 0.34161 | 0.35220 |
| Distrito Federal | 100,056 | 6,900 | 0.84279 | 0.08183 | 0.08436 |
| Espírito Santo | 2,295,624 | 478,744 | 0.80726 | 0.25834 | 0.26635 |
| Goiás | 21,538,072 | 3,360,762 | 0.86295 | 0.18082 | 0.18643 |
| Maranhão | 7,758,352 | 403,145 | 0.78666 | 0.06605 | 0.06810 |
| Mato Grosso | 28,592,183 | 637,513 | 0.88853 | 0.02509 | 0.02587 |
| Mato Grosso do Sul | 21,003,830 | 429,911 | 0.89430 | 0.02289 | 0.02360 |
| Minas Gerais | 23,707,042 | 9,298,950 | 0.81867 | 0.47912 | 0.49398 |
| Pará | 19,911,217 | 477,386 | 0.80176 | 0.02990 | 0.03083 |
| Paraíba | 1,145,943 | 210,023 | 0.65128 | 0.28141 | 0.29013 |
| Paraná | 9,181,577 | 4,252,134 | 0.86119 | 0.53776 | 0.55443 |
| Pernambuco | 1,920,075 | 728,726 | 0.73364 | 0.51733 | 0.53336 |
| Piauí | 1,660,099 | 141,692 | 0.65986 | 0.12935 | 0.13336 |
| Rio de Janeiro | 2,379,648 | 527,468 | 0.78940 | 0.28079 | 0.28950 |
| Rio Grande do Norte | 972,816 | 315,487 | 0.69913 | 0.46386 | 0.47824 |
| Rio Grande do Sul | 13,956,953 | 4,299,228 | 0.81678 | 0.37713 | 0.38882 |
| Rondônia | 12,744,326 | 683,608 | 0.77630 | 0.06910 | 0.07124 |
| Roraima | 735,962 | 12,385 | 0.69528 | 0.02420 | 0.02495 |
| Santa Catarina | 4,285,931 | 2,687,826 | 0.79055 | 0.79328 | 0.81787 |
| São Paulo | 10,126,223 | 1,801,011 | 0.89543 | 0.19863 | 0.20478 |
| Sergipe | 1,218,972 | 355,306 | 0.82196 | 0.35462 | 0.36561 |
| Tocantins | 8,062,227 | 273,698 | 0.75781 | 0.04480 | 0.04619 |

**Map-based estimate of current yields of beef and milk on pasture**

We calculated the current yields of beef and milk per hectare using the production estimates from Equations 3 and 4, and cultivated pasture areas from LAPIG ([www.lapig.iesa.ufg.br/lapig](http://www.lapig.iesa.ufg.br/lapig)). This is an approximation, because some native pastures are not mapped by LAPIG, while others may be mapped as cultivated pastures. We assumed that all production was from pasture areas mapped by LAPIG. We used these numbers to estimate current yields per hectare for each pixel as follows:

${CY}_{b}=\frac{{CBP}_{t}}{CPA}$ **Eq. 3**

Where: CY*_b_* is the current yield beef in tonnes per hectare for each municipality, CBP_t_ is the current beef production in tonnes for each municipality and CPA is the total cultivated pasture area in hectares for each municipality.

${CY}_{m}=\frac{{CMP}_{t}}{CPA}$ **Eq. 4**

Where: CY*_m_* is the current yield milk in tonnes per hectare for each municipality, MP is the current milk production in tonnes for each municipality and CPA is the total cultivated pasture area in hectares for each municipality.

These yield estimates were converted to food energy units per hectare (GJ/ha) by multiplying CY*_b_* and CY*_m_*, by conversion factors of, respectively, 8.28432 GJ/t for beef and 2.55224 GJ/t for milk.

We also calculated the number of heads per hectare, and converted this to animal units per hectare, for comparison with the potential yields (**Eqs. 5 and 6**). To convert to animal units, we used state-level conversion factors (AU per head) calculated by Rafael Barbieri from a detailed analysis of herd composition in each state (Supplementary Table 1).

$CH=\frac{CH_{m}}{CPA}$ **Eq. 5**

Where: CH is the current cattle of heads per hectare per municipality; CH_m_ is the total of cattle heads in each municipality and CPA is the total cultivated pasture area in hectares in each municipality.

$C_{AU}=CH*{AU}_{s}$ **Eq. 6**

Where: C_AU_ is the current animal units per hectare per municipality, CH is the current cattle heads per hectare in each municipality and AUs is the total animal units per cattle head in each State.

**Calculation of additional potential production of beef and milk on pasture**

We calculated the additional potential to produce beef and milk on cultivated pasture in Brazil, using the estimates already described, plus maps of potential for pasture grass production and other data. We obtained projections of the potential production of rain-fed pasture grasses (RFPG) under different climate scenarios for the 2020s from the Global Agro-Ecological Zones (GAEZ) website (gaez.fao.org). We averaged these projections to get a single raster map with a mean projected value for each pixel. The map units were 10 kg dw/ha per year, and thus were converted to yields in kg dw/ha by multiplying all values by 10. We used equation 8 from (*1*) to convert these potential grass yields to stocking rate in Animal Units (AU), assuming a daily feed intake (I) of 8 kg/AU/d and grazing efficiency (E) of 50% (****Eq.** 7**). The Potential stocking rate is in the units AU/ha.

$P_{AU}=\frac{RFPG*10}{16*35}$ **Eq. 7**

Where: P_AU_ is the potential production in animal units per hectare per pixel; *RFPG* is the potential production of rain-fed pasture grasses from GAEZ in 10 kg dw/ha per year; 10 is the factor to convert from 10 kg/dw/ha per year to kg/dw yields; 16 is the feed intake per animal, incorporating the grazing efficiency of 50% (I/E in the terminology (*1*)); 365 is the factor to convert from annual dry matter yields (as given by GAEZ) to daily dry matter yields (as used in Equation 8 of (*1*)).

We clipped this map of potential to the LAPIG map of pasture, and calculated the mean value for each municipality (P_AU_).

Next, we calculated the difference (gap) between the potential stocking rates and the current stocking rate (**Eq. 8**). All units here are AU/ha:

${GAP}_{AU}= P_{AU}- C_{AU}$ **Eq. 8**

**Where: GAP_AU_ is the yield gap in animal units per hectare for each municipality; P_AU_ is the potential yield in animal units per hectare in each municipality and C_AU_ is the current yield in animal units per hectare in each municipality.**

We converted the gap in AU to heads (**Eq. 9**), using the state-level conversion factors from Supplementary Table 2.

${GAP}_{H}=\frac{{GAP}_{AU}}{{AU}_{s}}$ ****Eq. 9****

**Where: GAP_H_ is the yield gap in cattle heads per hectare in each municipality; GAP_AU_ is the yield gap in animal units per hectare in each municipality; and** AU_s_ is the total animal units per cattle head in the relevant State.

We converted this gap to beef equivalents (**Eq.10**) using the region-specific conversion factors established previously (Supplementary Table 1).

${GAP}_{B}={GAP}_{H}* B_{TH}$**Eq. 10**

Where: GAP_B_ is the beef yield gap in tonnes per hectare in each municipality; **GAP_H_ is the yield gap in cattle heads per hectare in each municipality; and** B_TH_ is the total cattle carcass weight produced divided by the total number of cattle heads in the region (not just those slaughtered) in tonnes.

To convert pasture yield gaps (and map-based estimates of current yields) from tonnes/ha to GJ/ha, we used a conversion factor of 8.28432 GJ/t for beef (GAP_B_GJ_).

We then used the mean yield gap and mean yield estimates, together with municipality-level production estimates, to estimate the additional production potential (*AddPot*, in GJ) of beef and milk in each municipality (**Eq. 11 and 12**).

${AP}_{B\_GJ}= {GAP}_{B\_GJ}*CPA$ **Eq. 11**

Where: AP_B_GJ_ is the additional potential of beef in GJ in each municipality; GAP_B_GJ_ is the beef yield gap in GJ per hectare in each municipality and CPA is the total cultivated pasture area in hectares in each municipality.

We calculate the additional potential of milk, using **Equation 12**.

${AP}_{M\_t}= {CMP}_{t}*\frac{{GAP}_{AU}}{C_{AU}}$ **Eq. 12**

Where: AP_M_t_ is the additional potential production of milk in tonnes in each municipality; CMP_t_ is the current milk production in each municipality in tonnes; **GAP_AU_ is the yield gap in animal units per hectare in each municipality and** C_AU_ is the current animal units per hectare in each municipality.

To convert pasture yield gaps (and map-based estimates of current yields) from tonnes/ha to GJ/ha, we used a conversion factor of 2.55224 GJ/t for milk (GAP_M_GJ_).

This results in an estimate of the additional potential production, for each municipality. Then, we summed the values for additional potential for beef and milk to get an overall estimate of the additional potential for cattle production on cultivated pasturelands.

**Supplementary Table 3.** Conversion factors to convert tonne units to food energy in GJ. Source: National Nutrient Database for Standard Reference Release 28; U.S. Department of Agriculture, Agricultural Research Service. 2014. USDA National Nutrient Database for Standard Reference, Release. Nutrient Data Laboratory ( <http://www.ars.usda.gov/nutrientdata>).

| **Commodity** | **Food energy GJ/t crop** |
| --- | --- |
| Beef | 8.28432 |
| Cassava | 5.75718 |
| Maize | 15.27160 |
| Milk | 2.55224 |
| Millet | 15.81552 |
| Rice | 15.27160 |
| Sorghum | 13.76536 |
| Soybean | 18.66064 |
| Sugarcane | 1.58992 |
| Wheat | 14.30928 |

**Additional production potential for sugarcane and soybean on cultivated pasturelands**

Additional food could be produced by planting sugarcane or soybean on cultivated pasturelands, which might exceed their potential to produce livestock products. To quantify this potential, we first calculated the mean potentially achievable yield (in GJ/ha) for each of these crops in each municipality, based on Earth Stat maps and food energy conversion factors from the USDA National Nutrient Database (Supplementary Table 3). See the **Equations 13 and 14**.

${MPY}_{SugarcaneM}=\sum\frac{{MPY}_{SugarcaneP}}{NP}*1.58992$ **Eq. 13**

Where: MPY_SugarcaneM_ is the mean potential yield of sugarcane in GJ/ha in each municipality; MPY_SugarcaneP_ is the mean potential yield of sugarcane in tonne/ha for each pixel in a given municipality; NP is the number of pixels in the given municipality and 1.58992 is the conversion factor in GJ/t to convert tonnes of sugarcane to food energy.

${MPY}_{SoybeanM}=\sum\frac{{MPY}_{SoybeanP}}{NP}*18.66064$ **Eq. 14**

Where: MPY_SoybeanM_ is the mean potential yield of soybean in GJ/ha in each municipality; MPY_SoybeanP_ is the mean potential yield of soybean in tonne/ha for each pixel in a given municipality; NP is the number of pixels in the given municipality and 18. is the conversion factor in GJ/t to convert tonnes of soybean to food energy.

We then multiplied this figure by the total area of pastureland (in hectares) in that municipality. This gives an estimate in GJ for each municipality (Equation 15 and 16). Note that this calculation uses the mean potential yield (not the yield gap) and thus gives an estimate of total potential production (not additional potential). The mean is calculated only for those pixels which contain pasture, using the pasture map of LAPIG.

${PPSugarcane}_{GJM}={MPY}_{SugarcaneM}{*TPA}_{M}$ **Eq. 15**

Where: PPSurgarcane_GJM_ is potential production of sugarcane in GJ for each municipality; MPY_SugarcaneM_ is the mean potential yield of sugarcane in GJ/ha in each municipality; TPA_M_ is the total pasture area in each municipality.

${PPSoybean}_{GJM}={MPY}_{SoybeanM}{*TPA}_{M}$ **Eq. 16**

Where: PPSoybean_GJM_ is potential production of soybean in GJ for each municipality; MPY_SoybeanM_ is the mean potential yield of soybean in GJ/ha in each municipality; TPA_M_ is the total pasture area in each municipality.

We then subtracted current production of beef and milk, and multiplied the result by the proportion of pasture in each municipality that is cultivated, to give an estimate of the additional potential for sugarcane or soybeans on pasture to produce food energy, per municipality. There is uncertainty in this calculation, as yields are likely to be lower on natural grassland than on cultivated, but potential is likely to be higher on cultivated pasture as the most productive grasslands are those which are most likely to have been cultivated. It is not clear, therefore, whether the yield gap is greater or lower on cultivated pasturelands relative to natural grasslands. Our implicit assumption in this calculation is that the yield gap is similar on both. A further source of uncertainty is the extent to which the LAPIG map includes natural grasslands in addition to cultivated pastures.

${APSugarcaneC}_{GJM}={(PPSugarcane}_{GJM}-({BP}_{GJM}+{MP}_{GJM}))$ **Eq. 17**

Where: APSugarcaneC_GJM_ is the additional potential production of sugarcane in cultivated pasture areas in GJ for each municipality; PPSugarcane_GJM_ is the potential production of sugarcane in GJ in each municipality; BP_GJM_ is the beef production in GJ in each municipality and MP_GJM_ is the milk production in GJ in each municipality.

${APSoybeanC}_{GJM}={(PPsoybean}_{GJM}-({BP}_{GJM}+{MP}_{GJM}))$ **Eq. 18**

Where: APSoybeanC_GJM_ is the additional potential production of soybean in cultivated pasture areas in GJ in each municipality; PPSoybean_GJM_ is the potential production of soybean in GJ in each municipality; BP_GJM_ is the beef production in GJ in each municipality and MP_GJM_ is the milk production in GJ in each municipality.

We took the maximum value of the three estimates: APSugarcane_GJM_ in cultivated pasture areas, APSoybean_GJM_ in cultivated pasture areas and APCattle_GJM_ in cultivated pasture areas as an estimate of the additional potential using the most productive of the available options for cultivated pastures. We applied a version of Equation 13 to these results to estimate the potential contribution of these changes to food security, and then we again calculated the maximum of the three estimates (for soybeans, sugarcane and beef/milk) to give an estimate of the potential contribution of agricultural improvements to cultivated pasture for food security.

${APFSugarcaneC}_{GJM}={APSugarcaneC}_{GJM}*1.58992$ **Eq. 19**

Where: APFSugarcaneC_GJM_ is the additional potential production of sugarcane for food in cultivated pasture areas in GJ in each municipality; PPSugarcane_GJM_ is the potential production of sugarcane in GJ in each municipality and 1.58992 is the conversion factor in GJ/t to convert tonnes of sugarcane to food energy.

${APFSoybean}_{GJM}={APSoybeanC}_{GJM}*18.66064$ **Eq. 20**

Where: APFSoybeanC_GJM_ is the additional potential production of soybean for food in cultivated pasture areas in GJ in each municipality; PPSoybean_GJM_ is the potential production of soybean in GJ in each municipality and 18.66064 is the conversion factor in GJ/t to convert tonnes of soybean to food energy.

**Note on trends in the proportion of pasture that is cultivated**

The share of cultivated pasture has increased in every agricultural census. In 1970, cultivated pastures made up only 19% of the total, while in 2006 they reached 64%. One could expect that in 2014 this share will be 76.8% (linear projection, R^2^=0.98) or 76.9% (log projection, R^2^=0.98). This means that by using estimates from 2006, our estimates of the additional production potential on cultivated pasturelands are likely to be conservative.

**Supplementary Table 4.** Proportion of total area of pasture which was identified as “cultivated”, between 1970 and 2006. The data were derived from agricultural census.

| Year | cultivated/total |
| --- | --- |
| 1970 | 0.192893342 |
| 1975 | 0.239666929 |
| 1980 | 0.34729174 |
| 1985 | 0.413499921 |
| 1996 | 0.560786406 |
| 2006 | 0.639887238 |
|  | |

**Supplementary Table 5.** Area, production and potential production of seven major crops and of livestock products from pasture in Brazil, in the reference year of 2015. More detailed data and sources provided in Supplementary Excel 1.

| **Product** | **Area (ha)** | **Total production (GJ)** | **Contribution to domestic food supply (GJ)** | **Additional production potential - total (GJ)** | **Additional production potential - contribution to domestic food supply (GJ)** |
| --- | --- | --- | --- | --- | --- |
| Rice | 2,162,178 | 187,859,021 | 152,682,162 | 78,718,594 | 64,476,678 |
| Sugarcane | 10,161,622 | 1,190,271,615 | 264,182,441 | 325,208,636 | 72,180,509 |
| Cassava | 1,536,161 | 132,758,867 | 52,878,501 | 45,669,595 | 18,190,422 |
| Maize | 15,846,517 | 1,302,433,153 | 169,875,067 | 404,059,712 | 52,701,109 |
| Soybean | 32,206,387 | 1,818,758,083 | 311,673,287 | 216,251,726 | 37,058,192 |
| Sorghum | 740,622 | 29,409,003 | 3,373,869 | 2,503,063 | 287,157 |
| Wheat | 2,490,115 | 78,821,968 | 78,821,968 | 9,942,046 | 9,942,046 |
| Total (seven crops) | 65,143,602 | 4,740,311,709 | 1,033,487,296 | 1,082,353,374 | 254,836,113 |
|  |  |  |  |  |  |
| Beef |  | 61,480,294 | 49,968,025 | 79,556,608 | 64,659,524 |
| Milk |  | 92,187,559 | 79,309,946 | 95,111,688 | 81,825,605 |
| Total (pasture) | 175,365,808 | 153,667,853 | 129,277,971 | 174,668,296 | 146,479,791 |
|  |  |  |  |  |  |
| Total | 240,509,410 | 4,893,979,563 | 1,162,765,266 | 1,257,021,669 | 401,315,904 |

**Technical assistance:** we used data from the IBGE’s Agricultural Census of the year 2006 (<https://sidra.ibge.gov.br/Tabela/791>) to calculate the percentage of farmers that receives some type of technical assistance. The IBGE data show the numbers of farmers with three classes of technical assistance: (i) occasionally receive, (ii) regularly receive and (iii) do not receive technical assistance. We calculated the percentage of farmers in each class, combining classes i and ii as farmers who receive assistance.

**Land tenure:** we used data from the IBGE’s Agricultural Census of the year 2006 (<https://sidra.ibge.gov.br/tabela/1244>) to calculate the percentage of legal status of the land. The IBGE data show the numbers of farmers on five classes of status of land: (i) landowner, (ii) land grant, (iii) renter, (iv) partnership and (v) occupant. We calculated the percentage of properties in each class, and we summed the percentage of farms with relatively secure land tenure (classes i, ii and iii) and the percentage of farms with less secure tenure (classes: iv and v).

**Percentage of native vegetation:** to estimate the percentage of native vegetation in each municipality we combined different data sources with coverage of each vegetation domain (for details of map source see the Supplementary Table 6). We converted all files to binary rasters with 30 meters of spatial resolution and calculated the percentage of native vegetation within each municipality boundary.

**Percentage coverage of Protected Areas and Indigenous Land:** we used the shapefile from the Environmental Ministry and FUNAI for the years 2016-2017 (<http://mapas.mma.gov.br/i3geo/datadownload.htm>; <http://www.funai.gov.br/index.php/shape>) to calculate the percentage of Protected Areas and Indigenous Land within each municipality boundary.

**Supplementary Table 6.** Sources of the data used to estimate the percentage of native vegetation in each Brazilian municipality.

| **Map** | **Reference** | **Source** |
| --- | --- | --- |
| Amazon | PRODES 2014 | <http://www.dpi.inpe.br/prodesdigital/prodes.php> |
| Atlantic Forest | SOS Mata Atlântica 2012-2013 | <http://mapas.sosma.org.br/> |
| Caatinga | Projeto de Monitoramento do Desmatamento dos Biomas Brasileiros por Satélite (PMDBBS) – 2008 - 2009 | <http://siscom.ibama.gov.br/monitora_biomas/PMDBBS%20-%20CAATINGA.html> |
| Cerrado | Terra Class Cerrado 2013 | <http://www.dpi.inpe.br/tccerrado/index.php?mais=1> |
| Pampa | LabGeo UFRGS 2007 (*4*) | <https://www.ufrgs.br/labgeo/index.php/dados-espaciais/246-mapeamento-da-cobertura-vegetal-do-bioma-pampa> |
| Pantanal | Projeto de Monitoramento do Desmatamento dos Biomas Brasileiros por Satélite (PMDBBS) – 2008 - 2009 | <http://siscom.ibama.gov.br/monitora_biomas/PMDBBS%20-%20PANTANAL.html> |

**Size of small, medium and large rural properties:** to characterize the size of rural properties according to fiscal modules we used the data available at: <http://www.imaflora.org/atlasagropecuario/> for the year 2017. For more details about the estimation of rural properties size see (*2*).

**Labour availability:** we used data from the IBGE’s Demographic Census of the year 2010 to estimate labour availability as described in **Equation 1**. Using the IBGE data, we selected the number of people more than 10 years old and classified in the category “economically active population” (<https://sidra.ibge.gov.br/tabela/1572>). The IBGE data separate rural and urban populations (<https://sidra.ibge.gov.br/Tabela/1378>). To estimate the total agricultural production area we used the OTIMIZAGRO map of the 2013 year (*1*) available here: <http://maps.csr.ufmg.br/>. We summed the area in hectares of all map classes associated with agriculture or livestock production.

$LA=\frac{EAP*\left( \frac{RP}{TP} \right)}{TPA}$ **Eq. 1**

Where: LA is the Labour Availability estimated per municipality in workers per hectare of agricultural land; EAP is the economically active population per municipality; RP is the total of rural population per municipality; TP is the sum of rural and urban population per municipality and TPA is the total of the areas used to agriculture or livestock on each municipality in hectares.

**Debt of Forest Code compliance:** we estimated the forest code compliance using the debt of PPAs (Permanent Preservation Areas) and LR (Legal Reserves) for the 6383 municipalities available on <http://maps.csr.ufmg.br/> , for more details about the debt calculation see (*3*). To match the debit estimation for our 5572 municipalities we converted the original debt map to a raster file and generated a central point in each municipality polygon to calculate the mean of PPAs and LR debit of each point.

**Surplus of Forest Code:** we obtained the surplus of natural vegetation from: <http://maps.csr.ufmg.br/>. For more details see (*3*). We matched the different definition of municipalities used there to ours using the same approach as described in the above item **Debt of Forest Code compliance**.

**Net Forest Code surplus:** here we used the **Debt of Forest Code** and the **Surplus of Forest Code** to estimate the **Net Forest Code surplus**. Firstly, we summed the debt of PPAs and LR. Then we subtracted the debt from the surplus.

**Supplementary Table 7.** Land area and additional production potential in quadrants and sub-categories.

| **Quadrant/sub-category** | **Number** | **(%)** | **Area**  **(M ha)** | **(%)** | **Additional production potential (total PJ)** | **(%)** | **Additional production potential (food PJ)** | **(%)** |
| --- | --- | --- | --- | --- | --- | --- | --- | --- |
|  |  |  |  |  |  |  |  |  |
| **Low priority** | **596** | **11%** | **67.39** | **8%** | **70.06** | **6%** | **27.77** | **7%** |
|  |  |  |  |  |  |  |  |  |
| **Increase yields** | **567** | **10%** | **43.60** | **5%** | **281.50** | **23%** | **71.73** | **18%** |
| Middle left | 272 |  | 27.90 |  | 122.53 |  | 30.83 |  |
| Upper left | 295 |  | 15.70 |  | 158.97 |  | 40.90 |  |
|  |  |  |  |  |  |  |  |  |
| **Protect and restore habitats** | **1693** | **30%** | **393.56** | **46%** | **148.05** | **12%** | **63.81** | **16%** |
| Lower mid | 903 |  | 116.59 |  | 87.97 |  | 35.12 |  |
| Lower right | 790 |  | 276.97 |  | 60.08 |  | 28.69 |  |
|  |  |  |  |  |  |  |  |  |
| **Municipality-level land-sparing policies** | **2714** | **49%** | **345.73** | **41%** | **741.26** | **60%** | **238.01** | **59%** |
| Middle mid | 568 |  | 58.66 |  | 172.07 |  | 44.38 |  |
| Middle right | 544 |  | 85.19 |  | 89.22 |  | 35.65 |  |
| Upper mid | 732 |  | 43.77 |  | 305.75 |  | 89.49 |  |
| Upper right | 870 |  | 158.11 |  | 174.22 |  | 68.49 |  |
|  |  |  |  |  |  |  |  |  |
| **Total** | **5570** |  | **850.28** |  | **1240.87** |  | **401.32** |  |

**References**

1. B. Soares-Filho *et al.*, Brazil’s market for trading forest certificates. *PLoS One* **11**, 1–17 (2016).

2. V. Guidotti *et al.*, Números detalhados do novo Código Florestal e suas implicações para o PRAs. *Sustentabilidade em Debate* **5**, 1–10 (2017).

3. B. Soares-filho *et al.*, Cracking Brazil ’ s Forest Code. *Science* **344**, 363–364 (2014).

4. H. Hasenack *et al.*, Remanescentes de Vegetação dos Campos Sulinos (do Pampa). *Ministério do Meio Ambient. Secr. Biodeversidade e Florestas*, 31 (2007).

5. B. B. N. Strassburg *et al.*, When enough should be enough: Improving the use of current agricultural lands could meet production demands and spare natural habitats in Brazil. *Glob. Environ. Chang.* **28**, 84–97 (2014).
